# *BMC Ecology* image competition: the winning images

**DOI:** 10.1186/1472-6785-13-6

**Published:** 2013-03-22

**Authors:** Simon Harold, Yan Wong, Michel Baguette, Michael B Bonsall, Jean Clobert, Nick J Royle, Josef Settele

**Affiliations:** 1 BioMed Central, Floor 6, 236 Gray’s Inn Road, London WC1X 8HB, UK; 2 Sue Rider Management, London, UK; 3 Institut de Systématique, Evolution et Biodiversité, Muséum National d’Histoire Naturelle (MNHN), UMR 7205, Paris, 75005, France; 4 Dept of Zoology, University of Oxford, Oxford, OX1 3PS, UK; 5 CNRS, Station d’Ecologie Expérimentale du CNRS à Moulis, USR 2936, Saint Girons, France; 6 Centre for Ecology and Conservation, Biosciences, College of Life and Environmental Sciences, University of Exeter, Cornwall Campus, Penryn, Cornwall, TR10 9EZ, UK; 7 Department of Community Ecology, Helmholtz Centre for Environmental Research-UFZ, Theodor-Lieser-Str. 4, Halle, 06120, Germany

## Abstract

*BMC Ecology* announces the winning entries in its inaugural Ecology Image Competition, open to anyone affiliated with a research institute. The competition, which received more than 200 entries from international researchers at all career levels and a wide variety of scientific disciplines, was looking for striking visual interpretations of ecological processes. In this Editorial, our academic Section Editors and guest judge Dr Yan Wong explain what they found most appealing about their chosen winning entries, and highlight a few of the outstanding images that didn’t quite make it to the top prize.

## Editorial

“*The camera industry is one of the few innocuous parasites in wild nature*” [1]

In his 1949 collection of essays *A Sand County Almanac* the ecologist Aldo Leopold lamented the loss of wild nature in his native US and the increasing disconnection between man and the natural world. Although he saw the use of photography as a means to reconnect people to ecosystems, would he ever have envisaged how ubiquitous the camera would become in the digital age?

The ease with which nature can now be captured using modern photography is manifest in many high-quality prizes established for professionals and amateurs alike; for example the Natural History Museum/ BBC Worldwide Wildlife Photographer of the Year [2], or the National Geographic Photo Contest [3].

While these showcase the undoubted talents of a diverse demographic of photographers, here at *BMC Ecology* we wanted to find out whether the natural world might be viewed differently from the perspective of professional ecologists like Leopold, with a specific emphasis on the central idea behind the study of ecology as a science—how organisms interact with each other and their environment.

Compared to many other scientists, ecologists might consider themselves fortunate. Although ecological processes are complex, they often have strikingly visual components: a bee pollinating a flower, a predator hunting its prey, a fight between two rivals, the intricate habitat of a growing tree. Our intimate connection to our environment gives such images a visceral appeal. At its best, that appeal can be used to tell an ecological story, illuminate our understanding of nature, and highlight our investigations into it. By asking ecologists to send us their visual interpretations of the natural world—not just limited to photography—we hope to bring you a unique window into how the world around us is currently being investigated.

In the spirit of scientific peer-review, we asked the Editorial Board of *BMC Ecology* to judge not only the visual appeal of these entries, but how they communicated this complexity. Every entrant was also given the opportunity to have their images published by our partners at the Biology Image Library [4], an online collection of resources for scientifically reliable teaching and learning in biology and biomedicine. All images are subject to peer-review before inclusion in the library, and include full metadata for each file.

To judge the overall winners, we are delighted to have Dr Yan Wong [5], an evolutionary biologist who – through the slightly different medium of BBC television and radio – also tells stories about science in general, and biology in particular. We're also very pleased to be taking up his suggestion of a donation to the Wytham Woods Appeal Fund [6], and in a minor way, contribute to the continued maintenance of this outdoor ecological laboratory, which has proved such a fruitful resource for generations of ecologists.

From the outset, we were aiming to garner entries from all branches of ecology. Indeed, in addition to being visually compelling and well composed, we were drawn to pictures that reflected in some way the enormous breadth of our science. The way to depict that broadness of scope was, of course, a matter of scientific and artistic individuality – individuality which is patently evident when inspecting the pictures below. We hope you will agree that our final selections are worthy winners. While your aesthetic opinions may not exactly tally with ours, we confidently expect a correlation between our and our readers' rankings of a strength which would, in normal circumstance, merit publication.

## Overall winners

Looking through the entries was a fascinating journey into a thriving jungle of ecological research – all the more enjoyable because many of the images submitted were visually stunning. This wasn't simply a search for an amazing picture, however. Just as important were the ecological processes depicted. Ideally, images should immediately hint at one or more ecological processes, yet leave some hidden depths which open up on closer inspection.

**Figure 1:**
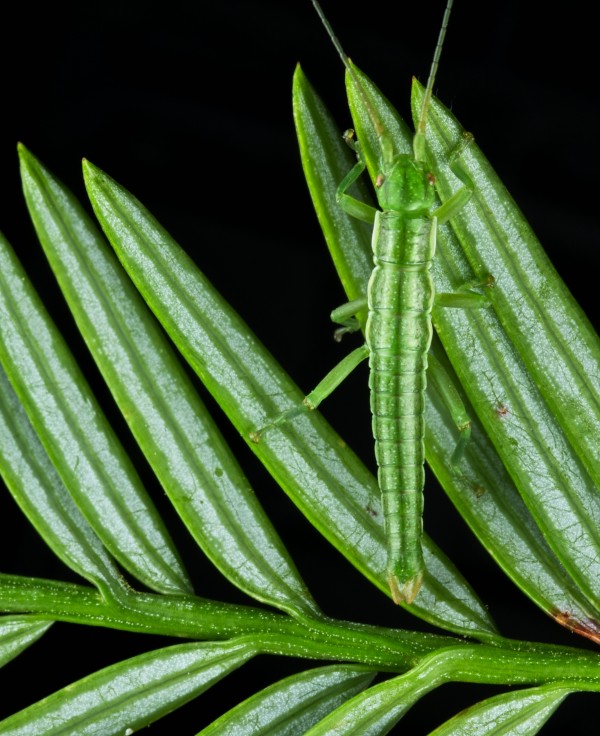
**Overall Winner. **“*Timema poppensis *perfectly camouflaged on its host, Redwood *Sequoia sempervirens, *California. This and other, closely related, species are adapted to live on very different host plants and at different elevations. These ecological specialisations have triggered the splitting into distinct species. How this ecological speciation is promoted, for example by divergent camouflage, can be studied by comparing species of *Timema *stick insects. Analysis of their DNA also reveals which regions in the genome play important roles in ecological speciation. The results of this research will advance our understanding of how biodiversity forms generally.” Attribution: Moritz Muschick.

The large number of highly commended images shows just how many ecologists have an eye for combining beauty with ecological insight. Five or six in particular gave the winning entries a run for their money. But in the eventual winner we recognised an extra quality: not only are the colours and composition eye-catching, they are the very basis of the underlying ecology – a dynamic that is being played out at a number of different levels and timescales. Even more impressively, this is revealed through the picture in such a clear way that it is immediately obvious, even to the layman. The image, taken by postdoctoral researcher Moritz Muschick from University of Sheffield, depicts the startling camouflage of a *Timema poppensis* stick insect against its host tree, the redwood *Sequoia sempervirens* (Figure 1).

From a purely visual point of view, the picture is striking: an almost geometrical arrangement whose two-toned green stripes stand in stark contrast to the pitch black background. This contrast only heightens the incredible visual similarity between the insect and the plant. The match is even more impressive considering that they come from very different branches of the tree of life.

The insect’s camouflage is broken somewhat by its orientation and an errant back leg, hinting that it is in the process of moving to an adjacent needle. We humans like reading this sort of dynamism and intentionality into a picture. In this case, however, it also serves as a counterpoint to ecological dynamic and an unconscious intentionality taking place on a rather different timescale – the millennia of adaptive change that have led to the evolution of this insect’s elegant colouration. A major factor in choosing this image as the winner is that it manages a seemly impossible task: to visualise an immensely long-term ecological process in a single static shot.

A moment’s thought reveals that the evolution of this camouflage may not be simple, because this *sequioa* is the insect’s host plant. While the insect benefits from its camouflage, the tree (presumably) suffers increased herbivory. This is ecological coevolution along the lines of Batesian mimicry. The accompanying text introduces the further topic of ecological speciation and generation of diversity—a topic of wide relevance to ecologists. And those are not the only hidden depths. At first glance, this looks like a picture which suffers in comparison to some other images in the competition, in that it involves only 2 species. There is however, a third player in the story – a role which we the viewers have assumed by simply glancing at the picture. It's as if we are looking at the scene through the very eyes of the predatory birds responsible for selecting this pigmentation. This is a winning picture because the viewer is, perforce, involved in the ecological process it depicts.

**Figure 2:**
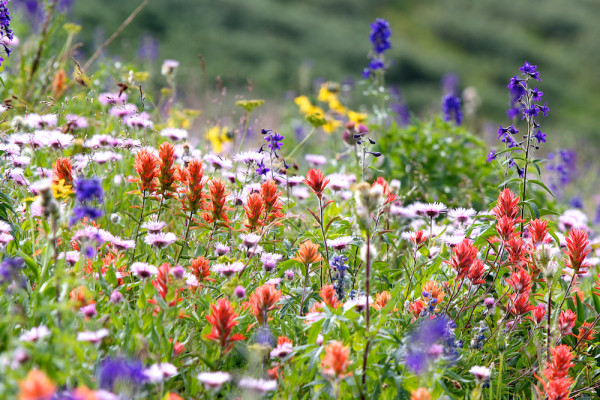
**Overall Runner-up. **“Multiple flower forms and phenologies visible in a subalpine meadow (Colorado)”. Attribution: Benjamin Blonder.

As a runner-up, we have selected a dazzling scene from Colorado of a subalpine flower meadow (Figure 2). Composing a photograph of this nature is surprisingly difficult, and Benjamin Blonder [7], a PhD student from University of Arizona, deserves congratulation for such a captivating portrayal of what it means to be biodiverse. The emphasis here is not on survival, but on reproduction: the dull but functional photosynthetic green seems an almost insignificant background compared to the waving of riotously coloured floral genitalia.

Although it can be seen as a poster child for the beauty of our science, it is only when we force ourselves to view the picture through an ecologist's eye that the true depths are revealed. What is it that allows such a diversity of forms and colours to coexist in an otherwise similar patch of ground? To whom are these flowers advertising, and what does this scene look like through their eyes? Like the winning image, the ecological emphasis of this photograph is on vision. But there the similarity ends. The similarity between the visual systems of humans and birds does not obviously extend to insects, the intended receivers of these plants' signals. We should perhaps be thankful that as unintended recipients, we can see beauty in this picture.

## Section winners

*BMC Ecology* is currently divided into several editorial sections by subject area, overseen by a group of academic Section Editors who were each asked to choose a winning image that best represented their specialist field of research. All entries were anonymized before being sent out for judging, and here some of the Editors outline why they feel these images were worthy winners of the category prize:

### Behavioural and physiological ecology

**Figure 3:**
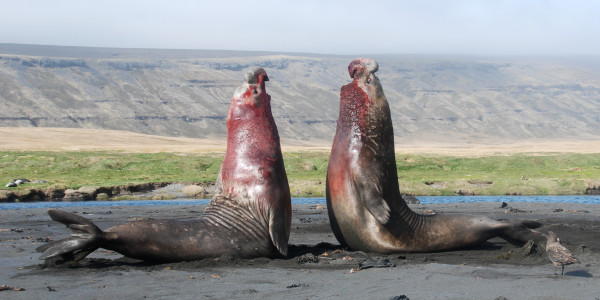
**Winner: *Behavioural and Physiological Ecology*. **“A harem of 127 females is a prize that has to be earned. Where female Southern Elephant Seals (*Mirounga leonina*) provide all the parental care, they will only reproduce in the territory of the biggest males, the biggest fathers for their offspring. Females average 400 to 900 kg, while males weigh up to 4 tons; Southern Elephant Seals show the largest sexual dimorphism among land breeding mammals. When a male challenges the head of a harem he needs to prove his strength at a great cost, resulting in a fascinating and captivating fight between the two giants.” Attribution: Laëtitia Kernaléguen.

This brutal photograph of two male Southern Elephant Seals (*Mirounga leonina*) fighting over a harem of 127 females was taken by Laëtitia Kernaléguen, a PhD student at Deakin University, Australia (Figure 3). Commenting on their choice of category winner, Section Editor Nick Royle felt that the photo—entitled “Is the prize worth the price?”—was:

“a terrifically vibrant shot of two giant scarred, bloody, Southern Elephant Seal males fighting for access to females. You can almost hear the roars of the combatants, and smell the blood, so well has this image captured the behaviour. I love the mirrored symmetry of the males as they engage in their violently ritualised, almost choreographed, fighting; the male on the right of the shot fixing his rival with a gimlet eye as he looks to land a decisive blow. The picture is very well composed, with the gore-covered males with their red bibs contrasting sharply with the serenely bleak but beautiful backdrop. A nice final touch is the skua in the bottom right of the frame, seemingly taking a keen interest in the outcome. Or perhaps (s)he is the referee?”

### Community, population and macroecology

**Figure 4:**
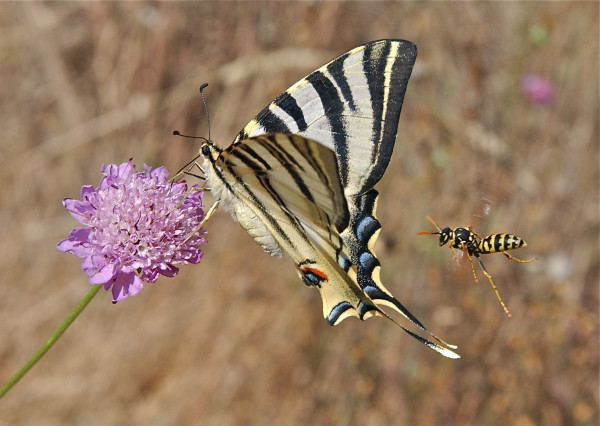
**Winner: *Community, Population and Macroecology*. **Scarce swallowtail, *Scabius *flower and Polistine wasp. Attribution: Michael Siva-Jothy.

This section considers research on the interaction of organisms within communities and populations across all scales, and it was a striking image of multitrophic interactions in action that Section Editor Jean Clobert felt captured the essence of this area of research. The photo (Figure 4), taken by Professor Michael Siva-Jothy from University of Sheffield (UK), was clearly not an easy one to take:

“I was taking pictures of scarce swallowtails on Scabius flowers when I heard a Polistine wasp buzzing around - It was hovering behind the butterflies and then darting in and body-checking them. Although I didn't witness predation I later saw a wasp dismembering a skipper in the same area. I’m pretty sure this wasp was trying to predate the swallowtail. I like the picture because it was difficult to take - the wasp was moving very fast and the focal plane was very shallow - and because it captures three trophic levels in one.”

### Conservation ecology and biodiversity research

**Figure 5:**
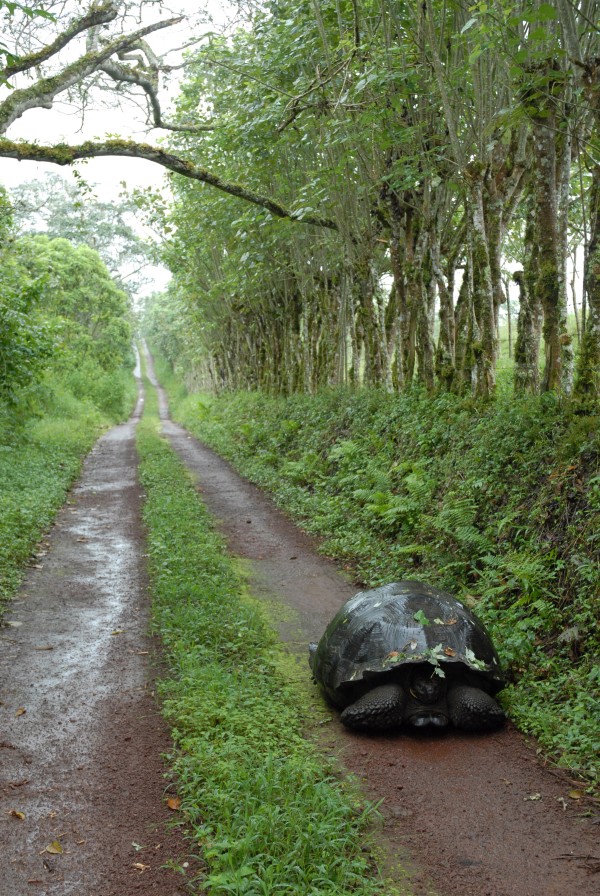
**Winner: *Conservation Ecology and Biodiversity.*** “A Galápagos tortoise (*Chelonoidis nigra*) utilizing a human road on Santa Cruz Island. I took this photograph while researching ecological interactions between species and landscapes in the Galápagos.” Attribution: Hara Woltz.

The Galápagos islands are something of a modern conservation symbol, due to the profound influence they had on the life and work of Charles Darwin. During his travels on the *Beagle*, Darwin noticed subtle anatomical differences between giant tortoises living on different islands in the archipelago, which would later inform his theory of evolution by natural selection. This image (Figure 5) by Hara Woltz [8], a conservation biologist from Columbia University (US), was taken while researching ecological interactions between species and landscapes in the archipelago, and depicts a Galápagos tortoise (*Chelonoidis nigra*) on Santa Cruz Island utilizing a human road. Section Editor Josef Settele found a subtle commentary behind the picture, which he felt might be renamed “Brother where are you bound?”:

“It shows nicely that a knowingly long-lived specimen of a low-density species does not necessarily seems to have a bright future, even though a rather large path is offered.”

### Landscape Ecology and Ecosystems

**Figure 6:**
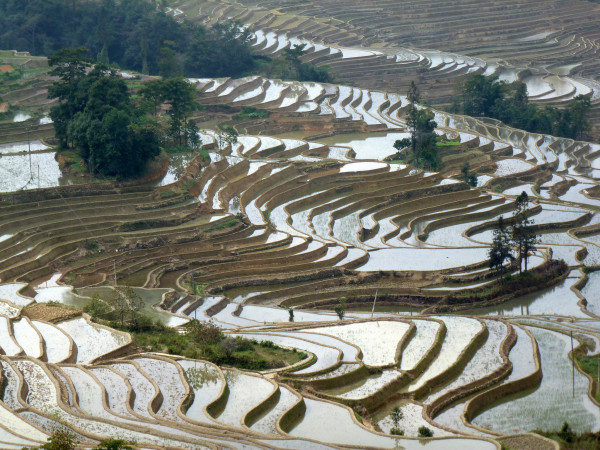
**Winner: *Landscape Ecology and Ecosystems*. **“Rice paddy in Yuanyang, China” Attribution: Yulin Jia. Biology Image Library ID 65539.

Depicting the spatial interactions between species and their influences on ecosystem processes, was one of the more challenging topics to capture visually. Section Editor Michel Baguette's selection was a wonderfully textured image of human-mediated landscape engineering. In this picture (Figure 6) [9] of a rice paddy in Yuanyang, China, research plant pathologist Yulin Jia from U.S. Department of Agriculture Dale Bumpers National Rice Research Center explains how rice paddies have been cultivated for more than 1000 years:

“Expansive areas of these terraced fields support enough rice for hundreds of thousands of people. Water is saved in the hilltop forests, and channelled down to the terraces for irrigation. The rice terraces are flooded from September to March, the evaporation from these paddies provides a cool and pleasant climate for local residents while the fields provide a spectacular view for travellers.”

### Theoretical ecology and models

**Figure 7:**
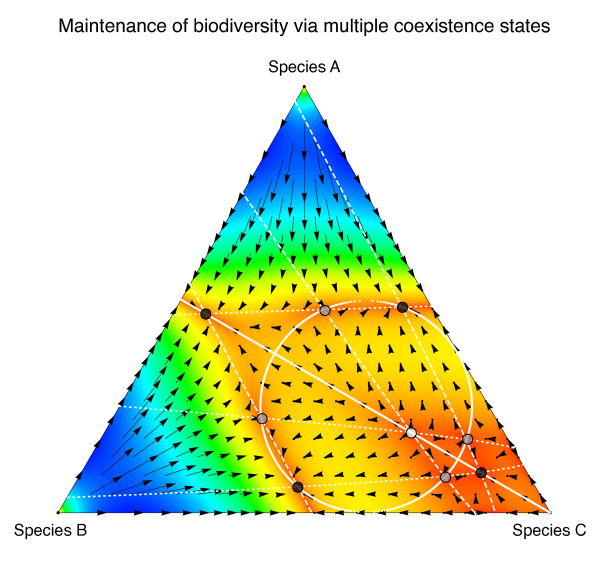
**Winner: *Theoretical Ecology and Models.*** Multiple players and the maintenance of biodiversity. From Gokhale CS, Traulsen A: Evolutionary games in the multiverse. *PNAS *2010, 107: 12, 5500-5504. (doi: http://dx.doi.org/10.1073/pnas.0912214107).

The section on theoretical models was always going to be one of the most challenging to depict visually, especially when judged alongside photographs of the natural world. It also presented perhaps the greatest opportunity for creativity. Data visualization is central to understanding the concordance of models with real-world data, and for a section that leant itself less to the camera, it represented an interesting opportunity for desk-bound ecologists to showcase their work. The winning entry chosen by Section Editor Michael Bonsall was submitted by Chaitanya Gokhale, a postdoctoral researcher at Max Planck Institute for Evolutionary Biology (Germany), and depicts a model of evolutionary game theory on the maintenance of biodiversity with multiple players (Figure 7). This figure is taken from their 2010 article in *Proceedings of the National Academy of Sciences* entitled “Evolutionary games in the multiverse” [10]:

“Evolutionary game theory can help us analyze interactions in a social or biological setting. My personal success depends not only on what I do but also what others do. In biological terms, being successful in an evolutionary game is positively correlated to the reproductive success. Typically, pairwise interactions are assumed in game theoretic interactions.

However in reality, interactions are not always in pairs. For example more than two hunters are often required if a big animal is to be taken down. Two player interactions predict that there can be at most a single point where the different strategies can agree on so that each gets an equal payoff and each strategy survives. If there are d players that can choose from n different strategies, then there can be at most (d-1)^(n-1) points where all the strategies can coexist. Thus, for d > 2, the number of such points increases exponentially with the number of strategies. Also the encounters between species can involve multiple individuals from the involved species. This provides the possibility for multiple stable states and thus eventual maintenance of biodiversity.

Thus, if there are multiple strategies involved and if the number of players is high, there may be higher chances of reaching a stable or unstable coexistence – simply because more solutions are possible.

The figure shows that if there are 4 players with 3 strategies (given by the vertices by the triangle) then there can be at most (4-1)^(3-1), that is, 9 points where the strategies have equal payoffs. These 9 points lie on the intersections of three cubic curves.”

### Editor’s Pick

**Figure 8:**
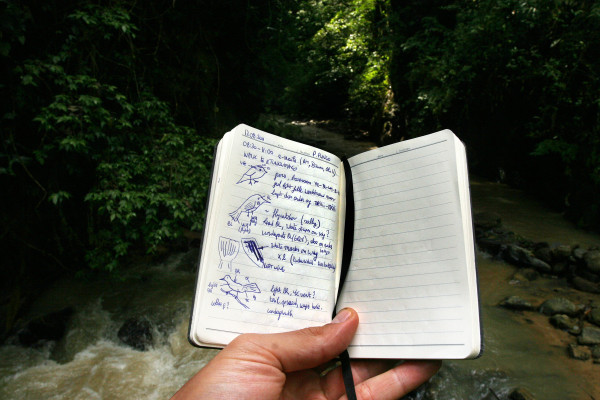
**Winner: *Editor's Pick.*** “Surveying old-growth secondary forest along the remote Rio Tunquimayo in the Puno province in SE Peru to determine the impact of coffee cultivation on bird conservation” Attribution: Raf Aerts.

The primary aim of this image competition was to achieve a sense of how professional ecologists visualize the natural world. While many of the stunning entries depicted ecological processes in action, few gave a sense of what it’s like to be an ecologist, to conduct research, to be driven to understand the natural world. Raf Aerts' image (Figure 8) stood out starkly by doing just that, by bringing the viewer directly into the eye of an ecologist on fieldwork, and represents one of the most creative uses of photography received this year. Here, Dr Aerts, a postdoctoral researcher from University of Leuven (Belgium) and University of Utah (US), explains a little of the background to this image, taken in an area of high-shade tree cover in the Peruvian Andes that is used to incentivize the conservation of forest biodiversity among coffee growers in the region:

“Here I am surveying old-growth secondary forest along the remote Rio Tunquimayo in the Puno province in SE Peru to determine the impact of coffee cultivation on bird conservation. The notebook shows my first record of the Yellow-browed Sparrow (above) and male and female White-winged Becards (mid and below). The sparrow may indicate disturbance, while the becard is a fly-catcher-like mid-level insect-eating bird of second growth and river-edge forest. The conservation of such insectivores is also beneficial for the coffee farmers as they contribute to insect pest control.”

### Highly commended

Although there could only be a handful of winning images in this year’s competition, the standard of entries was so high that there could easily have been several worthy winners across all categories. We do of course hope that you agree with our choices; and to give you a flavour of just how difficult these decisions were for all of our judges, here’s just a small selection of some of the most highly commended images that didn’t quite make it to the top prize.

Many of these images excel in specific ways, and in these particular aspects they are sometimes better than the winners we have selected. Take sheer visual beauty: the combination of colours in the image of an iridescent green bee [Additional file 1] [11] collecting bright yellow pollen from a purple *Vellozia* flower is surely one that will remain in the mind for some time to come. Indeed, one might have thought that the competition would be dominated by pictures of plant-pollinator interactions – relatively easy to capture images where colourful organisms are tied together via a clear ecological story. We are pleasantly surprised that this is not the case. In fact, only three such pictures make our final selection, and the other two are rather more unusual. One involves a vertebrate pollinator, in a winged blur of flower-powered energy [Additional file 2] [12]. The other shows that it sometimes takes an act of will to overcome our initial revulsion and appreciate the inherent beauty of an ecological scene [Additional file 3].

Several images show impeccable technical skill, such as the extraordinary picture of a hoverfly in mid flight [Additional file 4]. Indeed, this image was judged a close second place for the *Behavioural and Physiological Ecology* category prize, leaving Section Editor Nick Royle transfixed:

“*This image captures that brief moment of stillness – the hovering – of the fly perfectly; with just the blur of rapid wing beats the only movement. The fly clearly looks as if it is hovering, legs slightly dropped, perhaps to aid balance as it lands, or in readiness to shoot off again. This picture of nervous energy is greatly enhanced by the dazzling, iridescent colours and great detail; the eyes are so massive it looks like the fly is wearing a helmet*“.

Along with the similarly impressive winner of the *Community, Population and Macroecology* section (Figure 4), these surely would have been top in a standard competition of natural history photographs.

Almost as technically crisp are a pair of pictures that both tell behavioural stories: the gladiatorial standoff –or is it a polite discussion? between bulldog ants [Additional file 5] [13], and the simply delightful (albeit accidental) interaction between a duck and some frogs [Additional file 6].

Behavioural ecology leads to situations which make intriguing and hence captivating imagery. The Arabian babblers [Additional file 7] [14] are clearly up to something ecologically important, and we urge you to read the story of the ingeniously sonorous cricket [Additional file 8]. Multi-species interactions are represented by a crab and its biotic shelter [Additional file 9], although it's a shame we don't see it scavenging a livelihood on the surface of its travelling eco-home.

A less benign form of parasitism is seen in the colourful yet disturbing scene of death which must gladden the heart of foresters [Additional file 10]. Although death-by-wasp must be one of the most common of all gruesome endings in nature, we suspect only an ecologist would immediately recognise what is going on in the picture. The two other images of predators and their prey also take a while to fully absorb, giving the viewer an enjoyable moment of dawning realization. The diaphanous tentacles of a squid give this image a soft, aesthetic quality which sits in edgy contrast to the killing which has just taken place [Additional file 11]. The efficient dispatching of a bee by a well-hidden spider [Additional file 12] depends on exploiting an established plant-pollinator system: truly a multi-faceted portrait of ecology in action.

Five pictures concern human interaction with the environment, in a variety of guises. The image of a bird on the jawbone of a farm animal [Additional file 13] neatly and clearly captures a message which is all too familiar to conservation biologists. Attempts at understanding and communicating our impact on natural systems are shown in an elegantly simple picture of a blackboard [Additional file 14]. Ecologists interacting with their surroundings are represented here too. One serves as an awe-struck yardstick, emphasizing the sheer scale of the tree *Ceiba pentandra* [Additional file 15], while it takes a nice second or two to realize that a picture of nesting gannets is actually a portrait of practical conservation in action [Additional file 16]. Finally, the composition of contrastingly coloured conservationists [Additional file 17] is one of the most evocative descriptions of field ecology we have seen."

Looking to future competitions of this nature, there are some areas which seem somewhat unrepresented. There are few landscape photographs in our final selection, perhaps because it is hard to draw attention to a particular ecological detail when picturing a wide scene. The delightful exception to this is a nightscape dominated by luminous dinoflagellates [Additional file 18], whose anti-predator behaviour displays the stunning visual impact that ecology can sometimes provide. Perhaps the least well represented, however, are images which try to combine elements of photography and graphical display of information. The image of penguins and their heart rates [Additional file 19] (see also [15]) is a valiant attempt, but few entries attempted combinations of this ilk. Yet this sort of image gives perhaps the most scope for original and thought-provoking pictoral composition. It is surely a field worthy of more attention, if ecologists are to harness the power of visual impact to communicate their thoughts and research to a wider audience.

Individually these images provide a brief snapshot of ecology in action – sometimes brutal, sometimes beautiful. Collectively they provide an overview of complexity and connectivity in the natural world through which we hope readers will have been entertained―and a little enlightened.

### Notes

All images published in this Editorial are released under a Creative Commons Attribution License (CC BY) [16] by permission of the entrant. Please ensure you credit these names if you wish to re-distribute or re-use these fantastic pictures. You can also view other ecological images (including entries to the competition not listed here), on *BMC Ecology*’s Flickr page “Imaging Ecology” [17]. If you have some ecological images that you would like to share with us, please do join in.

## Competing interests

SH is an employee of BioMed Central. MB, MBB, JC, NJR and JS are Editorial Board Members for *BMC Ecology*. YW declares no competing interests.

## Authors’ contributions

SH conceived of the competition and co-wrote the Editorial with YW. MB, MBB, JC, NJR and JS chose category winners and provided quotations for their decisions. All authors read and approved the final manuscript.

## Supplementary Material

Additional file 1 “**Collecting pollen from *Vellozia*, Serra do Cipó, Brazil”. **Attribution: Daniel Wisbech Carstensen (Instituto de Biociências, Brazil). Biology Image Library ID: 64983. Click here for file

Additional file 2 **“A male Broad-tailed Hummingbird **(***Selasphorus platycercus***), **visits a scarlet gilia **(***Ipomopsis aggregata***) **flower at the Rocky Mountain Biological Laboratory, in Colorado. **These migratory hummingbirds fly from Mexico to Colorado each summer to reproduce, and are the primary pollinators of scarlet gilia flowers. Long-term studies of the phenology of the hummingbirds and the flowers they visit have been conducted since 1973 at RMBL, and show that the timing of both of these partners in the ecosystem service of pollination are changing, but not at the same rates. Males have an iridescent gorget, and produce a mechanical wing whistle that has a function in territorial displays (produced by the slot that is visible in this picture between the first two primary feathers). Taken with a Nikon D800e, 200 mm Nikkor macro lens, ISO640, 1/500 sec f10, SB800 flash.” Attribution: David W. Inouye. Biology Image Library ID 64660. Click here for file

Additional file 3 **“It’s a stinky feast! Blowflies just can’t resist the attraction of the orchid ** Captured during an orchid pollination observation in Kajang, Selangor, Malaysia, this photograph shows the orchid flowers of *Bulbophyllum lasianthum *Lindl surrounded by flies. During anthesis, these attractive purplish-red flowers emit a strong foul odour similar to that of carrion. The predominant species of flies found visiting and pollinating B. lasianthum was *Chrysomyia megacephala *Fabricius (Calliphoridae: Diptera), commonly known as blowflies. Both male and female blowflies are capable of pollinating the flowers of B. lasianthum. Some of the flies shown here actually have pollinia stuck on their backs as they happily work the flowers! Various fly species are extremely attracted to the flowers due to the stench as well as the carrion appearance of the flowers.”Attribution: Ong Poh Teck (Forest Research Institute Malaysia). Click here for file

Additional file 4 **) (Puerto Rico)” **Attribution: Benjamin Blonder (University of Arizona). Click here for file

Additional file 5 **“Communication in bulldog ants **(***Myrmecia nigriscapa,***) **Sydney, Australia” **Attribution: Sylvain Dubey (University of Lausanne). Biology Image Library ID: 65360. Click here for file

Additional file 6 “**The 2 frogs way enjoying the sun on the branch when the duck jumped up on the branch. **The frogs jumped for their lives!” Attribution: Thomas Jensen (Medical Prognosis Institute, Denmark). Click here for file

Additional file 7 **“Arabian babbler **(***Turdoides squamiceps***) **group allopreening in front of a neighboring group during a border confrontation.**” Attribution: Yitzchak Ben Mocha (Tel Aviv University). Image Library ID 63580. Click here for file

Additional file 8 “**This cricket was singing at dusk at the edge of secondary forest in Borneo.** He had crawled into the natural funnel of a ginger plant which was being used to amplify the sound of the song. I really liked the composition and decided I had to take it in natural light - this meant opening the aperture right up and slowing the shutter to 1/60. The effect was just what I wanted - a cricket with slightly blurred wings - capturing the movement - in a sea of blurred green with a strong sense of the funnel-nature of the plant. There are lots of pictures of singing crickets, but I know of none that capture this kind of behaviour - which is well-known.” Attribution: Michael Siva-Jothy (University of Sheffield). Click here for file

Additional file 9 **“A small crab, *Planes minutus* (Columbus crab), living on an individual of *Caretta caretta *(Loggerhead Sea Turtle). **This crab is known to prey upon other sea turtles epibionts.” Attribution: Maristella D'Addario (University of Rome). Click here for file

Additional file 10 “**Caterpillar of gypsy moth **(***Lymantria dispar***) **killed by the gregarious braconid wasp **(***Glyptapantheles liparidis***). This parasitoid is a major natural enemy of the worldwide known forest pest. 48 larvae developed in a single host and pupated under it after leaving the agonizing caterpillar’s body.” Attribution: György Csóka (Forest Research Institute Hungary). Click here for file

Additional file 11 “**With a refined and tenacious tactic of predation, the European squid **(***Loligo vulgaris***) **has captured a bream **(***Sparus aurata***) **launching its tentacles and applying a lethal bite in the prey column. **The image captures the moment when the squid seizes the prey with his arms.” Attribution: Miguel Cabanellas (Mediterranean Institute for Advanced Studies). Click here for file

Additional file 12 “**Multitrophic interactions in action” **Attribution: Anne Ebeling (University of Jena). Click here for file

Additional file 13 “**I think that this photo shows one of the most important issue in nature conservation nowadays. **And it is known because of habitat loss that a lot of species are facing big troubles to survive. So, people are trying to create reserves and refugees for animals. But the encroachment due to sprawling of cities highlights the problem of saving either one or another species, because in a restricted area often it is not possible to conserve all the species occurring inside. My photo is a way to underline this compromise.” Attribution: Matteo Lattuada (University of Antwerp). Click here for file

Additional file 14 **“Indirect gradient analysis is a powerful tool in community ecology to link species patterns to patterns of environmental variables including human disturbance.** During a field mission to Ethiopia, I gave an introduction to gradient analysis for researchers at Jimma University, with whom we investigate the effects of coffee cultivation on the diversity and community structure of epiphytic orchids, birds and trees in evergreen moist Afromontane forests.” Attribution: Raf Aerts (Univeristy of Leuven). Click here for file

Additional file 15 **“Rare large individual of *Ceiba pentandra *in lowland tropical forest” **Attribution: Benjamin Blonder (University of Arizona). Click here for file

Additional file 16 “**This image is of a constructed colony of decoy Northern Gannets on the North Island of New Zealand. **Calls are broadcast through solar powered speakers, and the decoys were set up to try to re-establish gannets on a preserved piece of land where Northern Gannets historically were found. I took this photograph while I was participating in a large scale ecological restoration project on this property.” Attribution: Hara Woltz (Columbia University). Click here for file

Additional file 17 “**Carrying vegetation survey equipment to a forest dynamics plot, Puerto Rico” **Attribution: Benjamin Blonder (University of Arizona). Click here for file

Additional file 18 “**Blue Tide. **Seasonal winds can cause the upwelling of nutrients which in turn can cause plankton populations to bloom as "red tides." Here, a dinoflagellate population (*Noctiluca *sp.) turns the ocean a luminous blue colour as the disturbance by the wind triggers a light-generating chemical reaction. The production of light is thought to attract fish predators that prey on potential predators of the dinoflagellates.” Attribution: Bruce Anderson (University of Stellenbosch). Click here for file

Additional file 19 “**To well understand all the ecological process that drive the physiology and behavior of animals in the nature, it appears really important to study organisms on the field. **It is also important for the scientists to estimate our impact during study on the free living species. Here, we measured the heart rate (HR) excess (the number of heart beats produced in excess of resting HR due to different kind of stress: capture, 10 m approach or sound). To do this, we used an externally mounted-HR logger (Polar® model RS800) on a free long-lived seabird, the king penguin (*Aptenodytes patagonicus*). We compared the HR response due to the same 3 stressors between two parts of the colony: one disturbed by human presence and one without humans. We show that the HR response is lower for the bird in the non disturbed place, for 10 m-approach and sound stress, but there is no difference between the two places for the stress of capture. Habituation of the king penguin or selection of the bird who can support the human proximity? The picture was taken in Crozet archipelago (46°24′41″S ; 51°45′22″E ), a French island of the austral ocean ; the 13th February 2012. The experimenter was positioning the HR logger on the back of the king penguin. On the right top of the image, we show an example of heart race trace we obtain and we can see clearly when the bird was caught for the stress of capture.” Attribution: Benoit Gineste (Evolutionary EcoPhysiology Group, CNRS). Click here for file
