# Supplementary figures and images for: BMC Ecology image competition: the winning images
Source: BMC Ecol. 2013 Mar 22;13:6. doi: 10.1186/1472-6785-13-6 (PMC3606310; doi:10.1186/1472-6785-13-6)

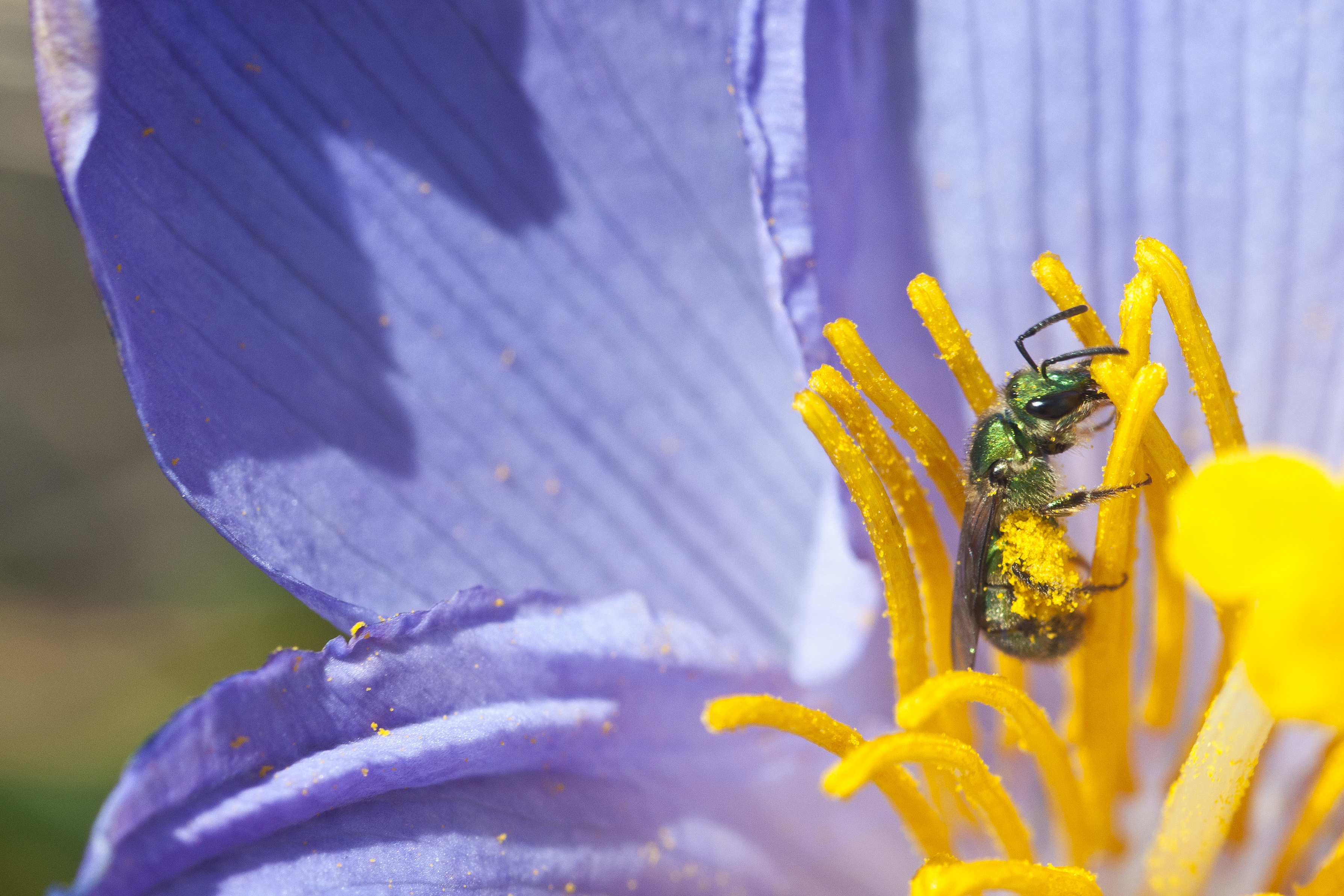

Supplement: Additional file 1 — “Collecting pollen from Vellozia, Serra do Cipó, Brazil”. Attribution: Daniel Wisbech Carstensen (Instituto de Biociências, Brazil). Biology Image Library ID: 64983. [file 1472-6785-13-6-S1.jpeg]

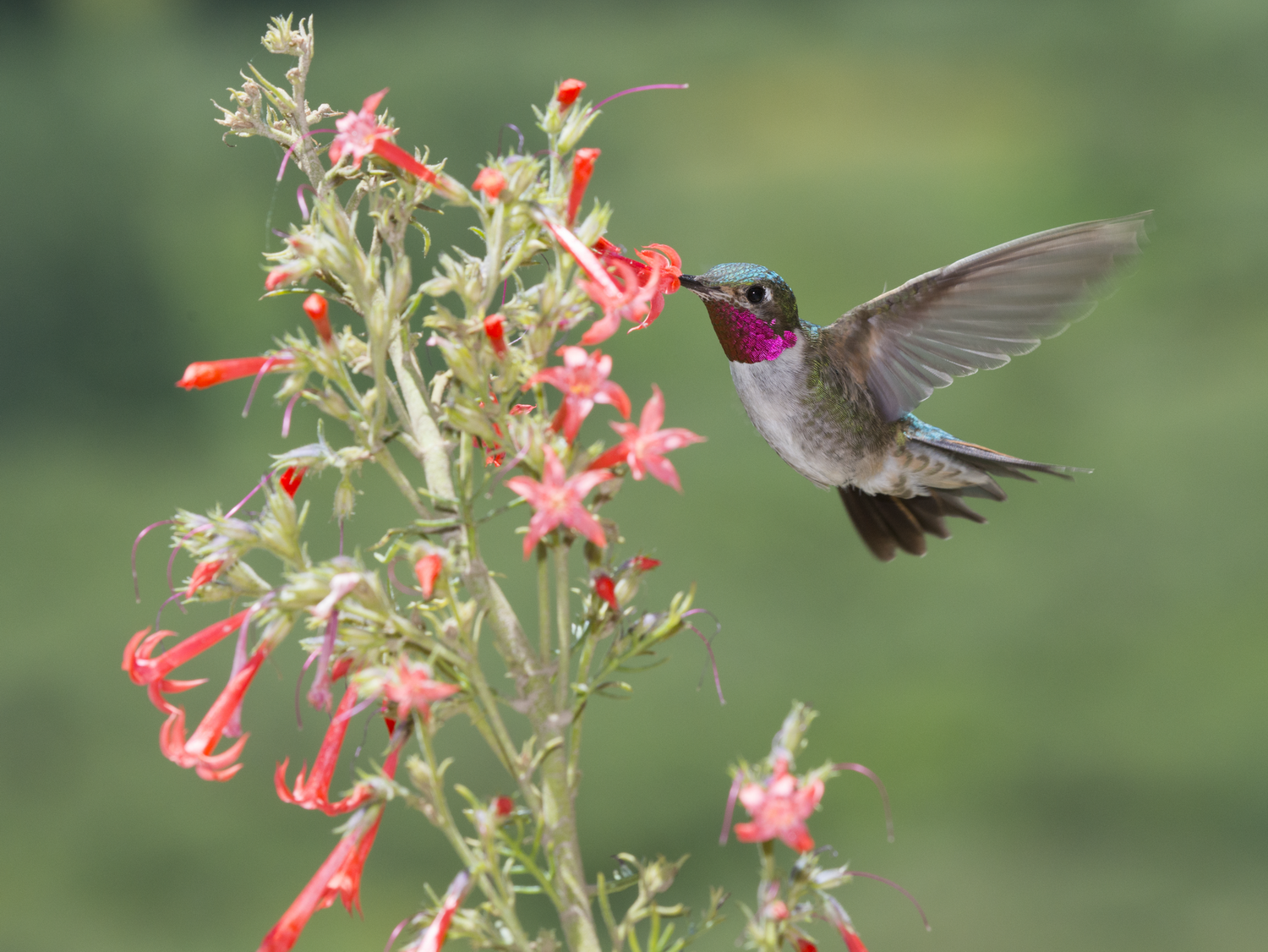

Supplement: Additional file 2 — “A male Broad-tailed Hummingbird (Selasphorus platycercus), visits a scarlet gilia (Ipomopsis aggregata) flower at the Rocky Mountain Biological Laboratory, in Colorado. These migratory hummingbirds fly from Mexico to Colorado each summer to reproduce, and are the primary pollinators of scarlet gilia flowers. Long-term studies of the phenology of the hummingbirds and the flowers they visit have been conducted since 1973 at RMBL, and show that the timing of both of these partners in the ecosystem service of pollination are changing, but not at the same rates. Males have an iridescent gorget, and produce a mechanical wing whistle that has a function in territorial displays (produced by the slot that is visible in this picture between the first two primary feathers). Taken with a Nikon D800e, 200 mm Nikkor macro lens, ISO640, 1/500 sec f10, SB800 flash.” Attribution: David W. Inouye. Biology Image Library ID 64660. [file 1472-6785-13-6-S2.tiff]

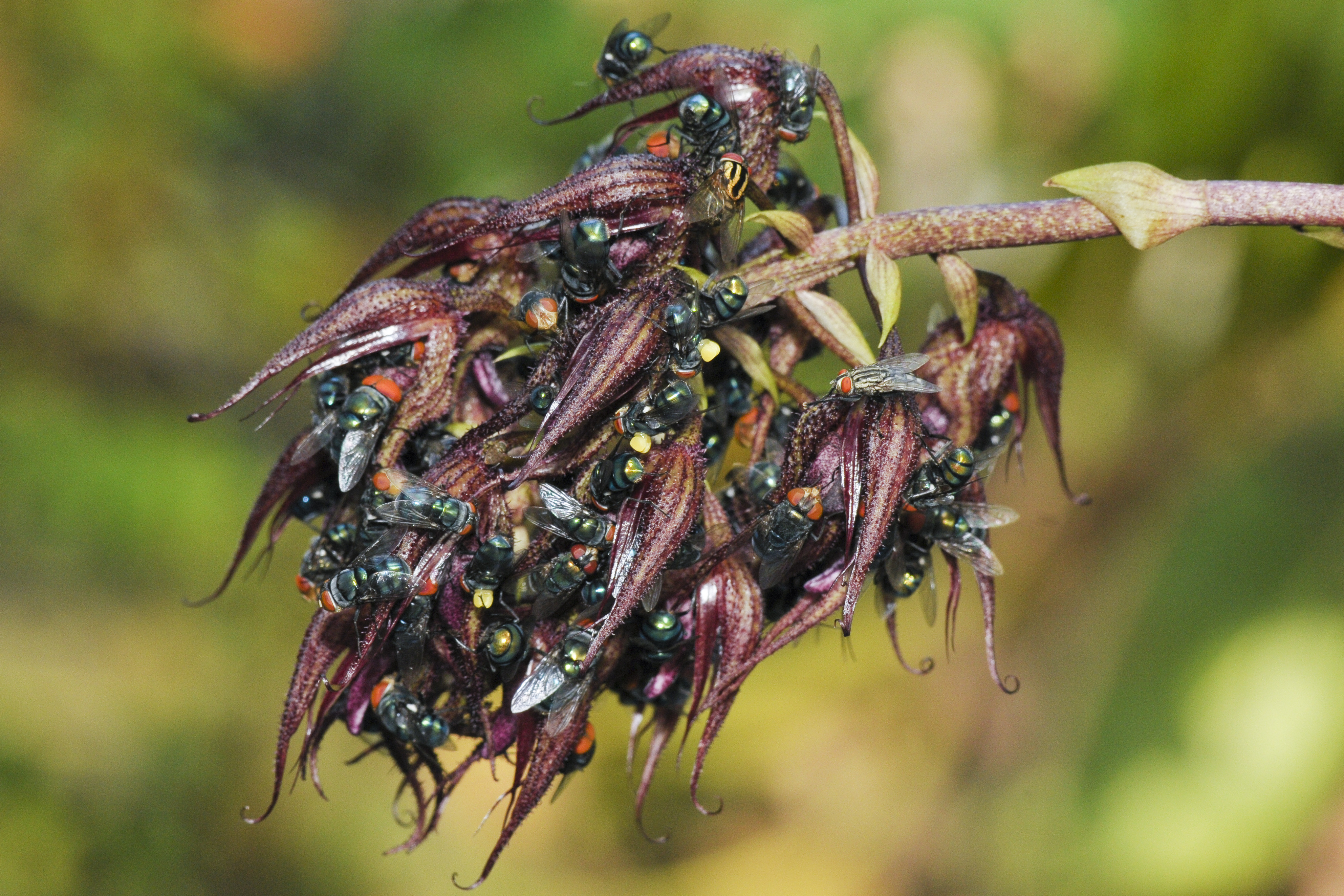

Supplement: Additional file 3 — “It’s a stinky feast! Blowflies just can’t resist the attraction of the orchid Captured during an orchid pollination observation in Kajang, Selangor, Malaysia, this photograph shows the orchid flowers of Bulbophyllum lasianthum Lindl surrounded by flies. During anthesis, these attractive purplish-red flowers emit a strong foul odour similar to that of carrion. The predominant species of flies found visiting and pollinating B. lasianthum was Chrysomyia megacephala Fabricius (Calliphoridae: Diptera), commonly known as blowflies. Both male and female blowflies are capable of pollinating the flowers of B. lasianthum. Some of the flies shown here actually have pollinia stuck on their backs as they happily work the flowers! Various fly species are extremely attracted to the flowers due to the stench as well as the carrion appearance of the flowers.”Attribution: Ong Poh Teck (Forest Research Institute Malaysia). [file 1472-6785-13-6-S3.jpeg]

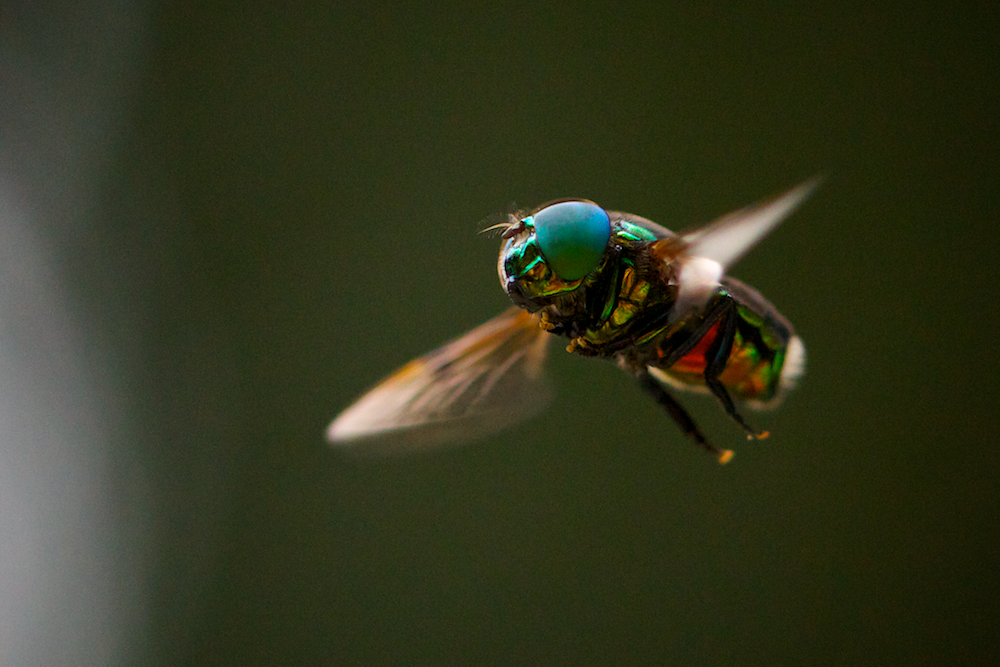

Supplement: Additional file 4 — ) (Puerto Rico)” Attribution: Benjamin Blonder (University of Arizona). [file 1472-6785-13-6-S4.png]

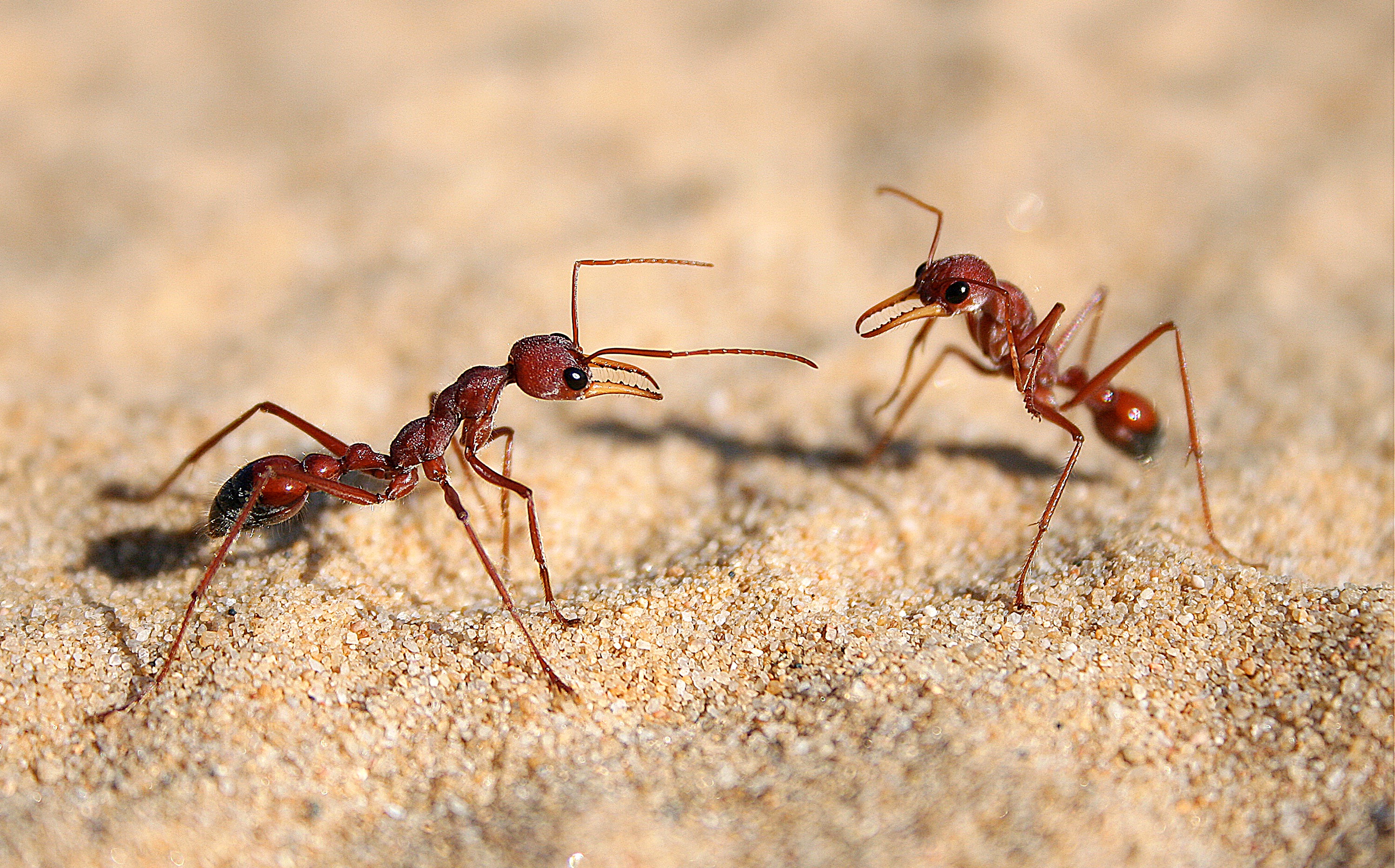

Supplement: Additional file 5 — “Communication in bulldog ants (Myrmecia nigriscapa,) Sydney, Australia” Attribution: Sylvain Dubey (University of Lausanne). Biology Image Library ID: 65360. [file 1472-6785-13-6-S5.jpeg]

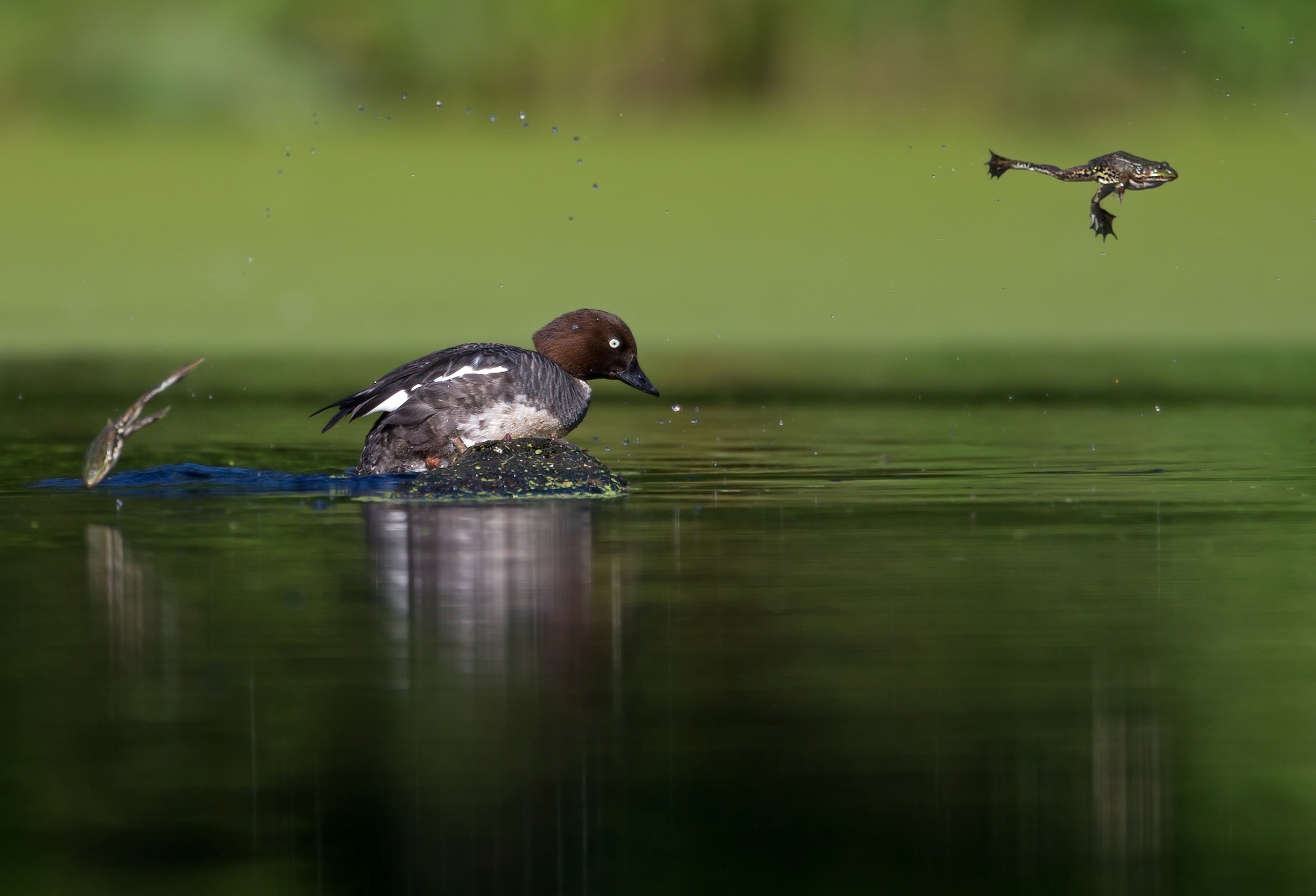

Supplement: Additional file 6 — “The 2 frogs way enjoying the sun on the branch when the duck jumped up on the branch. The frogs jumped for their lives!” Attribution: Thomas Jensen (Medical Prognosis Institute, Denmark). [file 1472-6785-13-6-S6.jpeg]

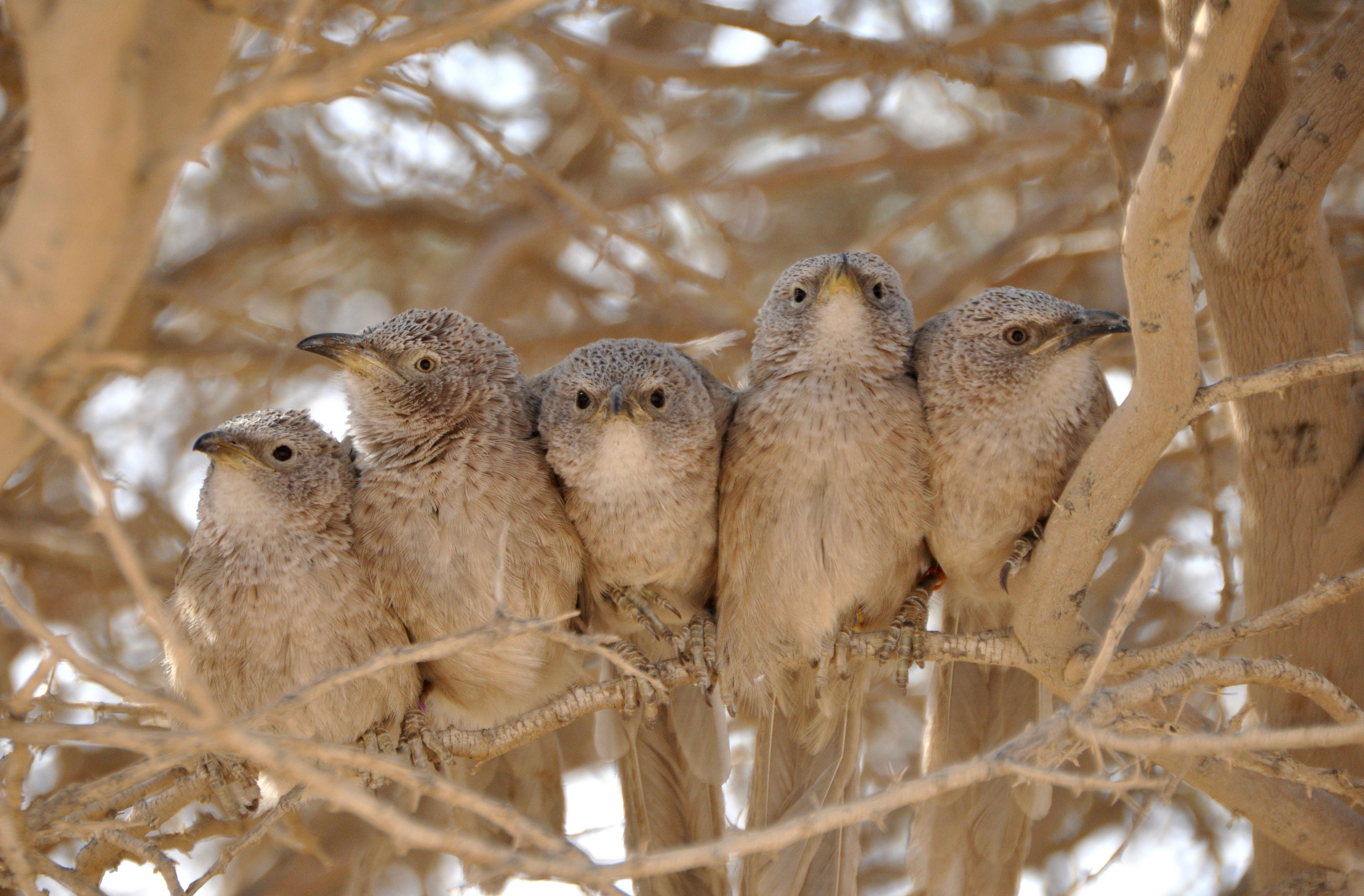

Supplement: Additional file 7 — “Arabian babbler (Turdoides squamiceps) group allopreening in front of a neighboring group during a border confrontation.” Attribution: Yitzchak Ben Mocha (Tel Aviv University). Image Library ID 63580. [file 1472-6785-13-6-S7.jpeg]

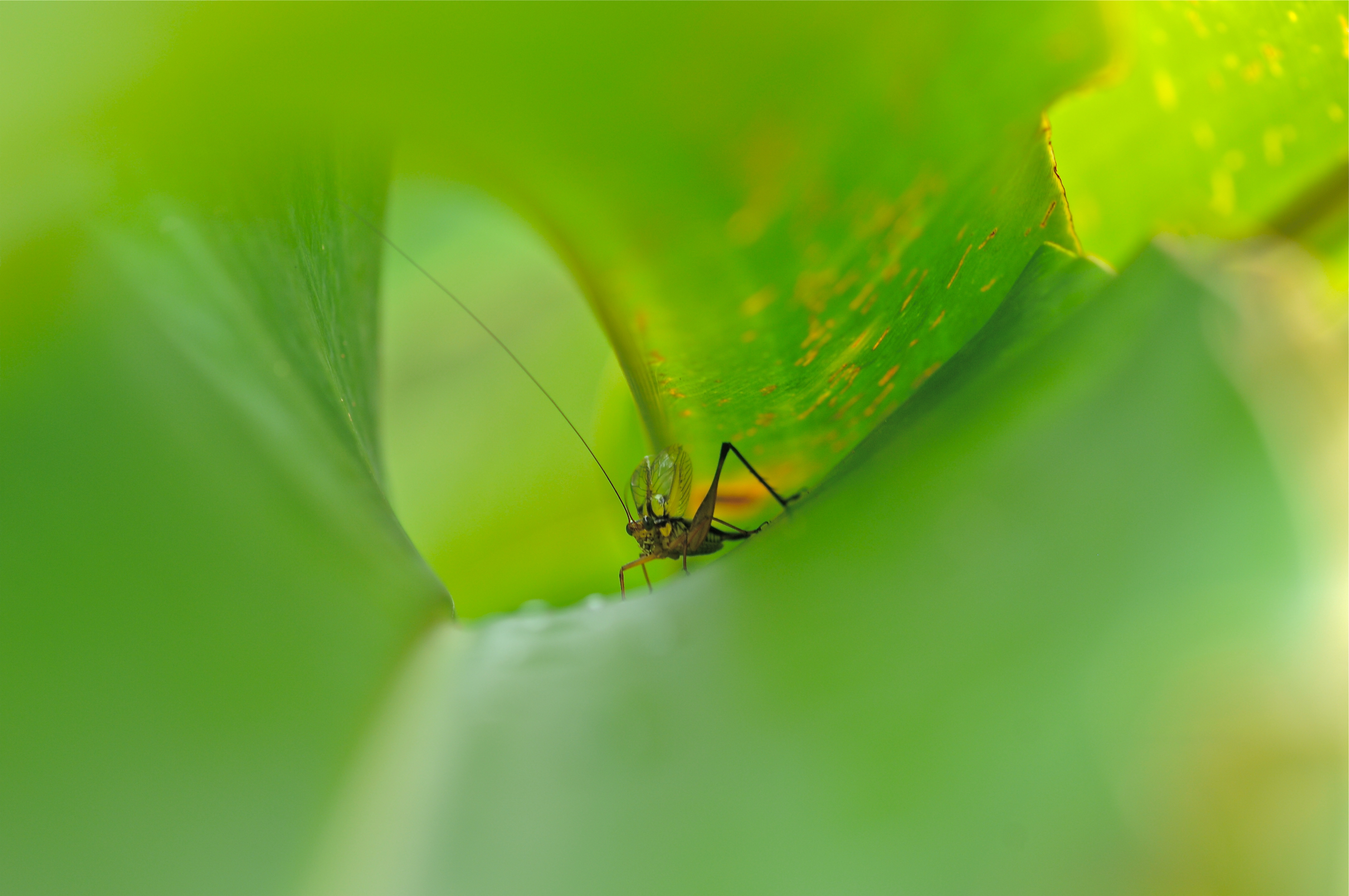

Supplement: Additional file 8 — “This cricket was singing at dusk at the edge of secondary forest in Borneo. He had crawled into the natural funnel of a ginger plant which was being used to amplify the sound of the song. I really liked the composition and decided I had to take it in natural light - this meant opening the aperture right up and slowing the shutter to 1/60. The effect was just what I wanted - a cricket with slightly blurred wings - capturing the movement - in a sea of blurred green with a strong sense of the funnel-nature of the plant. There are lots of pictures of singing crickets, but I know of none that capture this kind of behaviour - which is well-known.” Attribution: Michael Siva-Jothy (University of Sheffield). [file 1472-6785-13-6-S8.png]

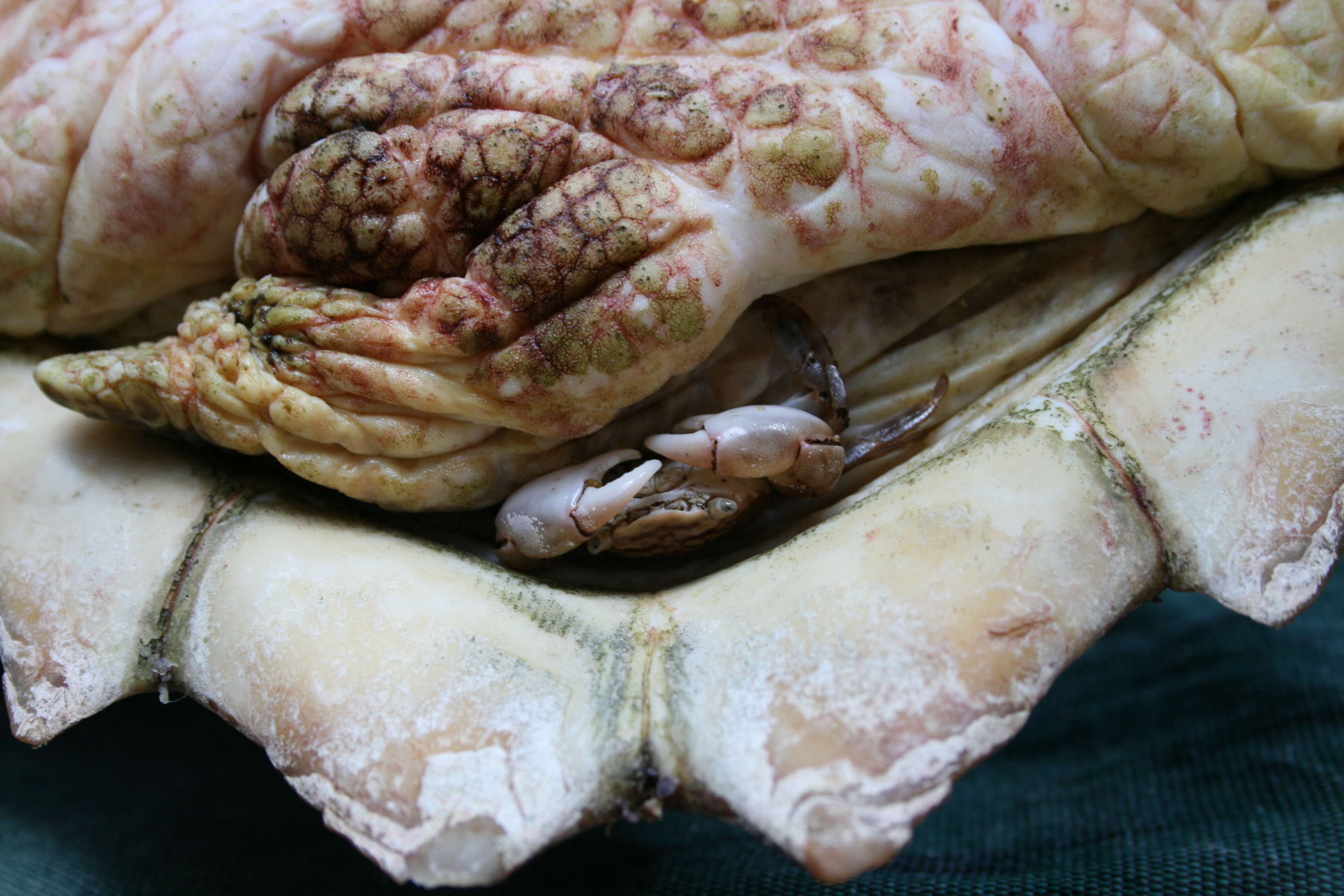

Supplement: Additional file 9 — “A small crab, Planes minutus (Columbus crab), living on an individual of Caretta caretta (Loggerhead Sea Turtle). This crab is known to prey upon other sea turtles epibionts.” Attribution: Maristella D'Addario (University of Rome). [file 1472-6785-13-6-S9.jpeg]

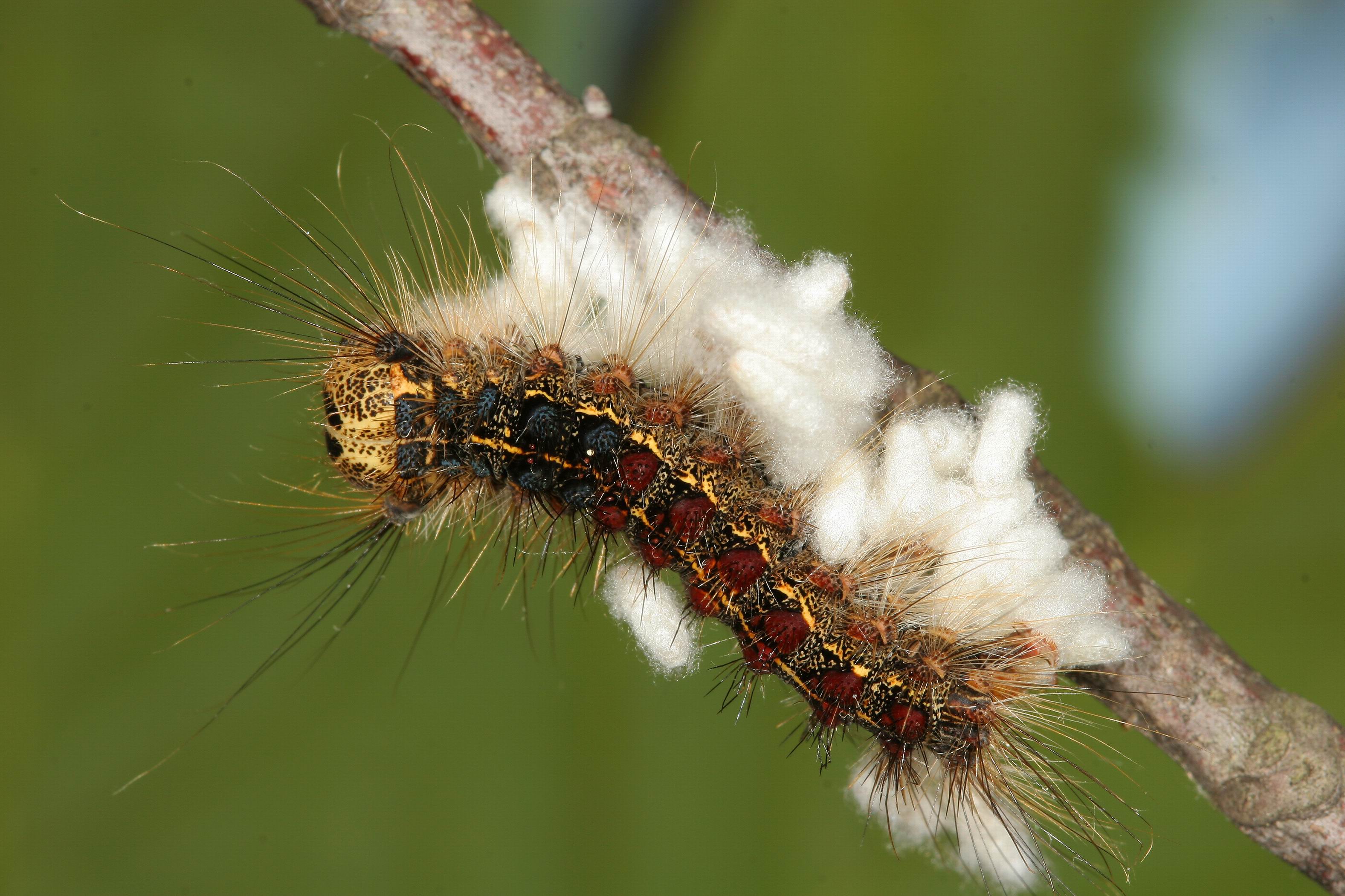

Supplement: Additional file 10 — “Caterpillar of gypsy moth (Lymantria dispar) killed by the gregarious braconid wasp (Glyptapantheles liparidis). This parasitoid is a major natural enemy of the worldwide known forest pest. 48 larvae developed in a single host and pupated under it after leaving the agonizing caterpillar’s body.” Attribution: György Csóka (Forest Research Institute Hungary). [file 1472-6785-13-6-S10.jpeg]

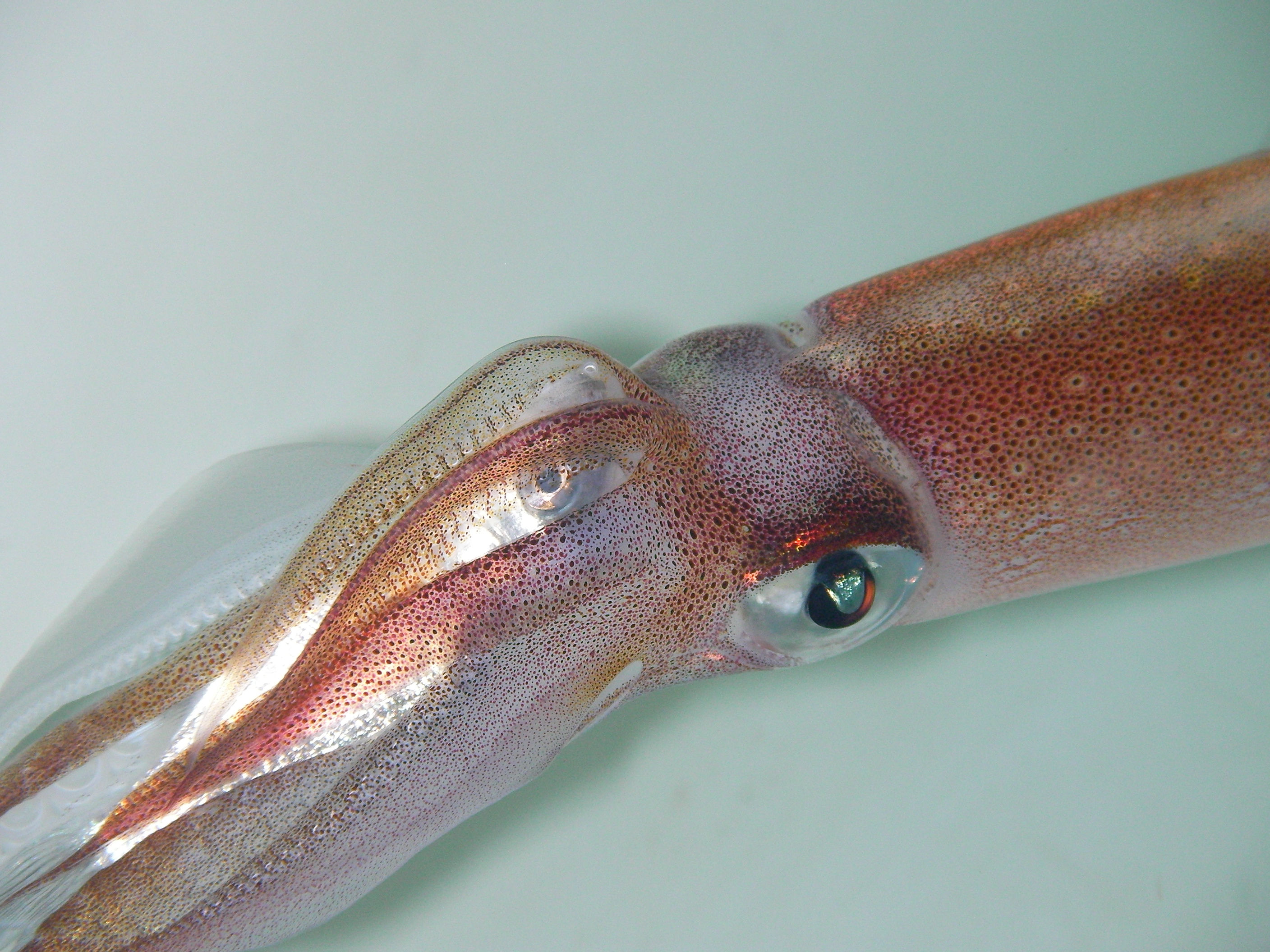

Supplement: Additional file 11 — “With a refined and tenacious tactic of predation, the European squid (Loligo vulgaris) has captured a bream (Sparus aurata) launching its tentacles and applying a lethal bite in the prey column. The image captures the moment when the squid seizes the prey with his arms.” Attribution: Miguel Cabanellas (Mediterranean Institute for Advanced Studies). [file 1472-6785-13-6-S11.jpeg]

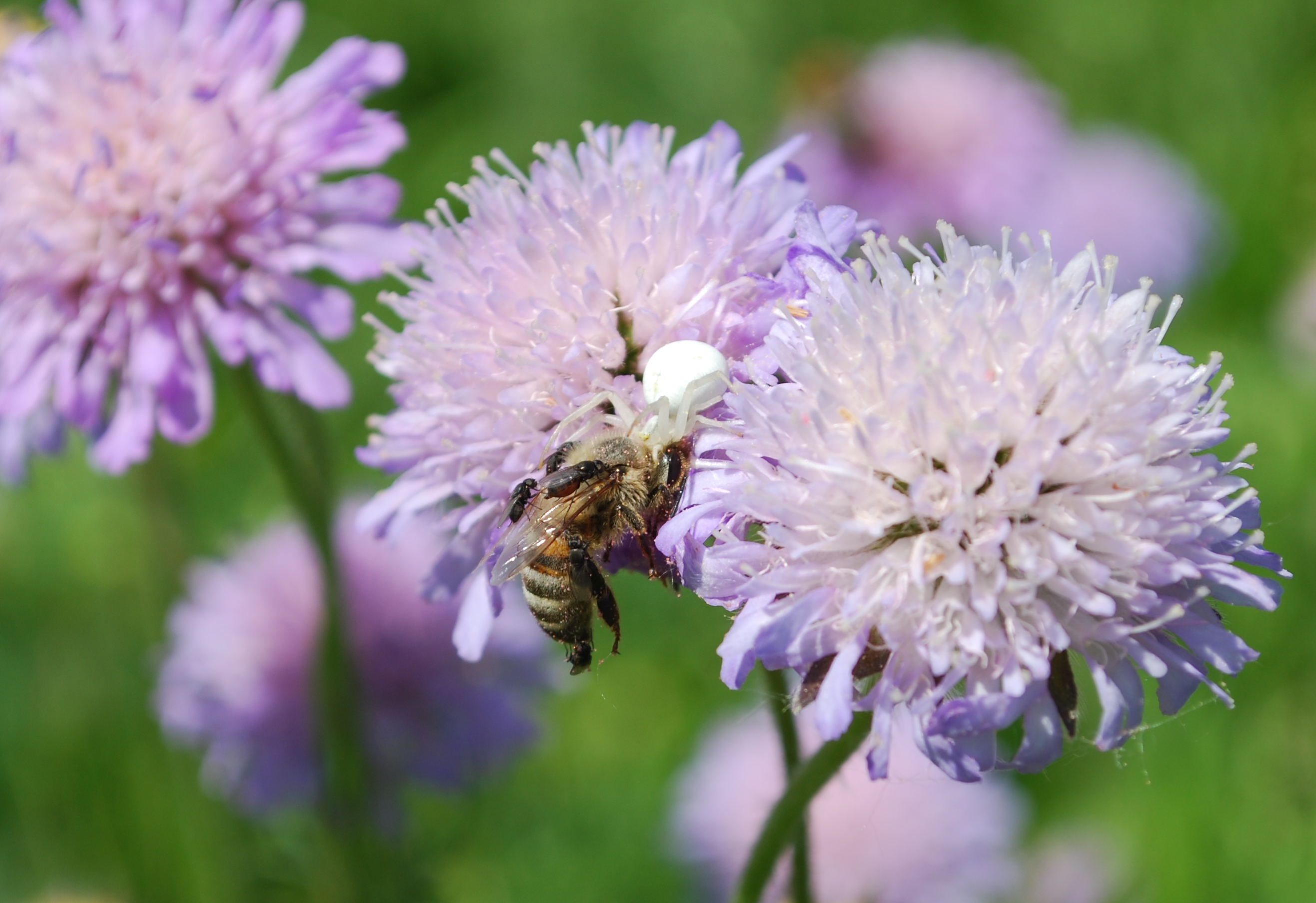

Supplement: Additional file 12 — “Multitrophic interactions in action” Attribution: Anne Ebeling (University of Jena). [file 1472-6785-13-6-S12.jpeg]

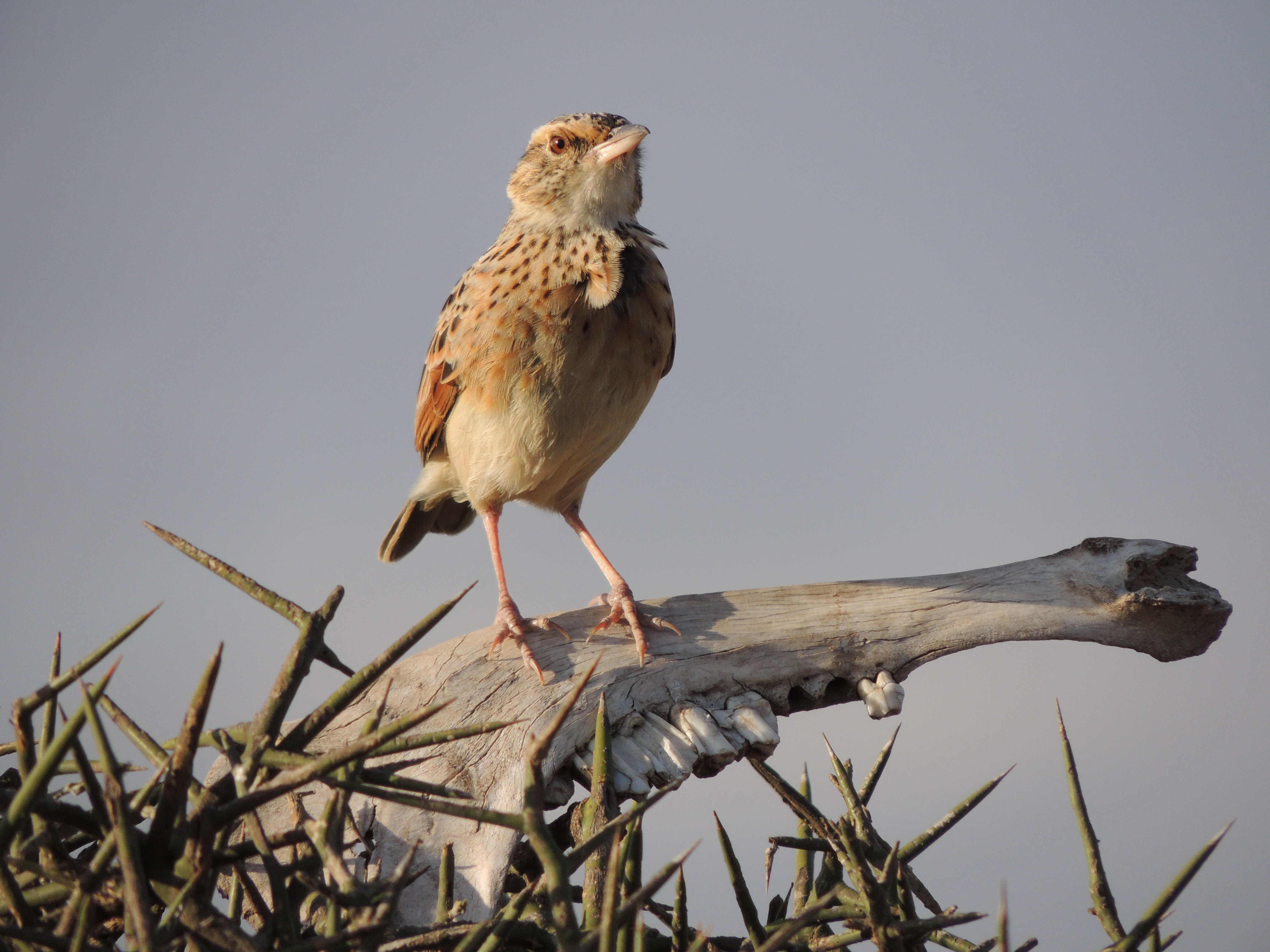

Supplement: Additional file 13 — “I think that this photo shows one of the most important issue in nature conservation nowadays. And it is known because of habitat loss that a lot of species are facing big troubles to survive. So, people are trying to create reserves and refugees for animals. But the encroachment due to sprawling of cities highlights the problem of saving either one or another species, because in a restricted area often it is not possible to conserve all the species occurring inside. My photo is a way to underline this compromise.” Attribution: Matteo Lattuada (University of Antwerp). [file 1472-6785-13-6-S13.jpeg]

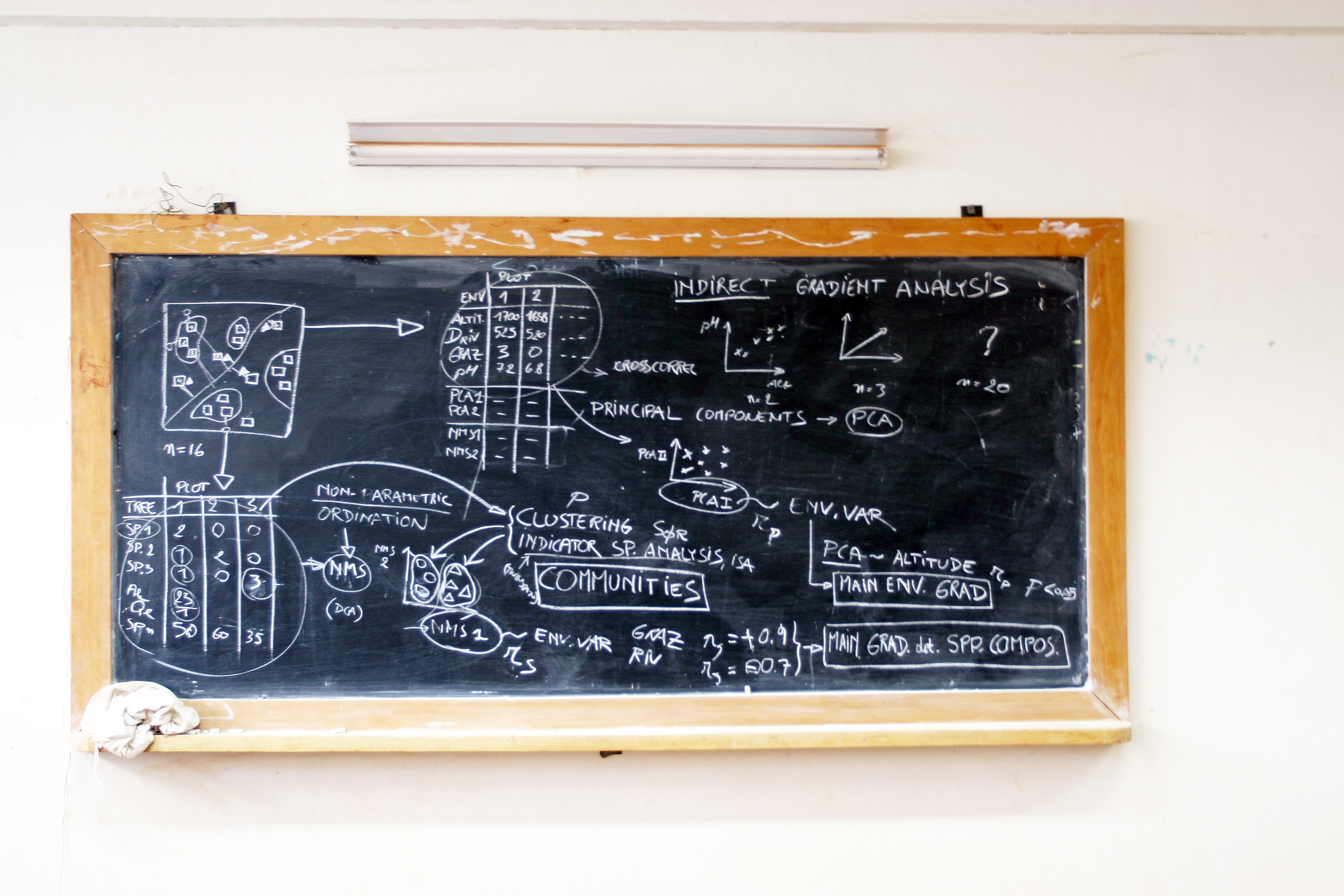

Supplement: Additional file 14 — “Indirect gradient analysis is a powerful tool in community ecology to link species patterns to patterns of environmental variables including human disturbance. During a field mission to Ethiopia, I gave an introduction to gradient analysis for researchers at Jimma University, with whom we investigate the effects of coffee cultivation on the diversity and community structure of epiphytic orchids, birds and trees in evergreen moist Afromontane forests.” Attribution: Raf Aerts (Univeristy of Leuven). [file 1472-6785-13-6-S14.jpeg]

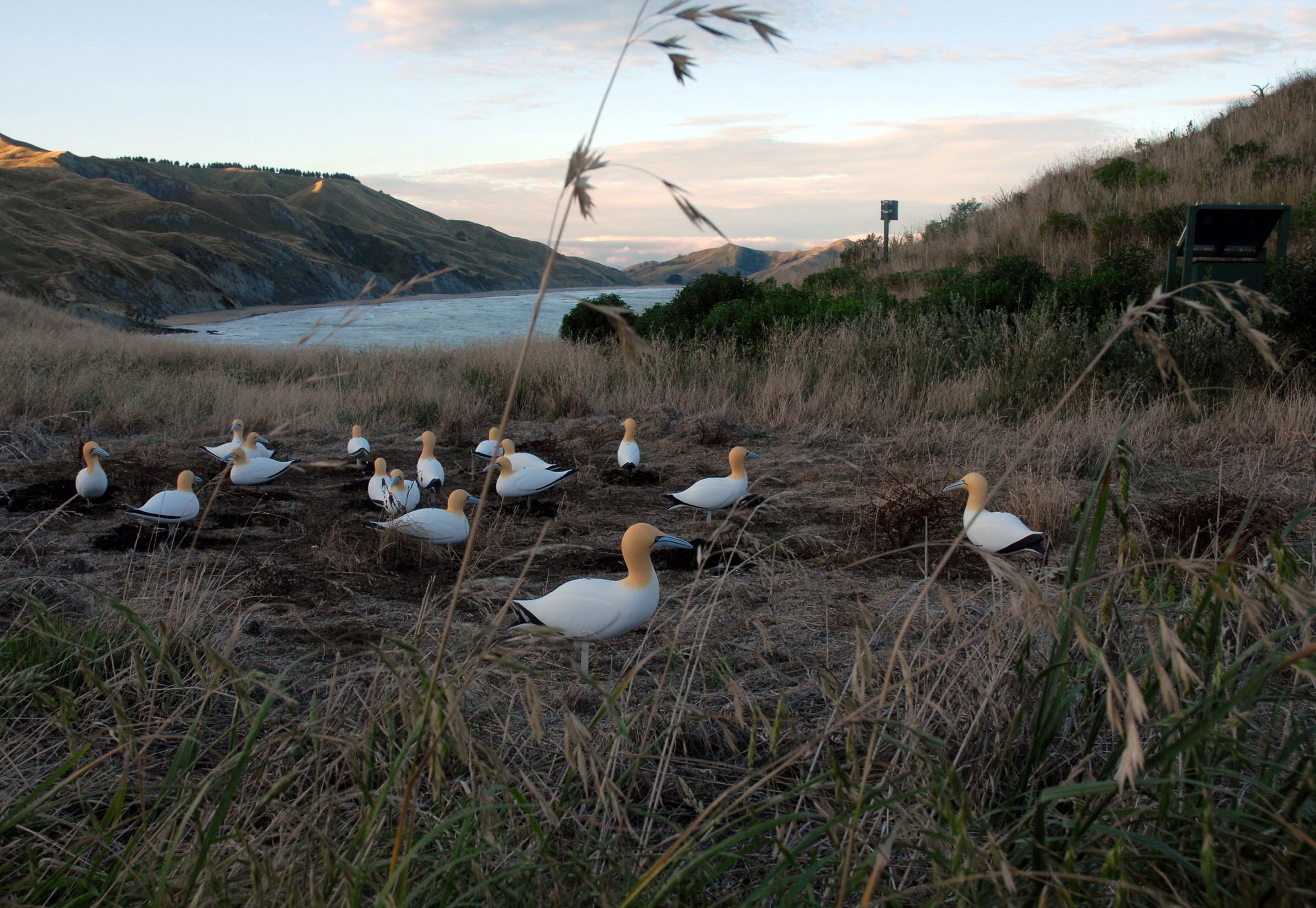

Supplement: Additional file 15 — “Rare large individual of Ceiba pentandra in lowland tropical forest” Attribution: Benjamin Blonder (University of Arizona). [file 1472-6785-13-6-S15.jpeg]

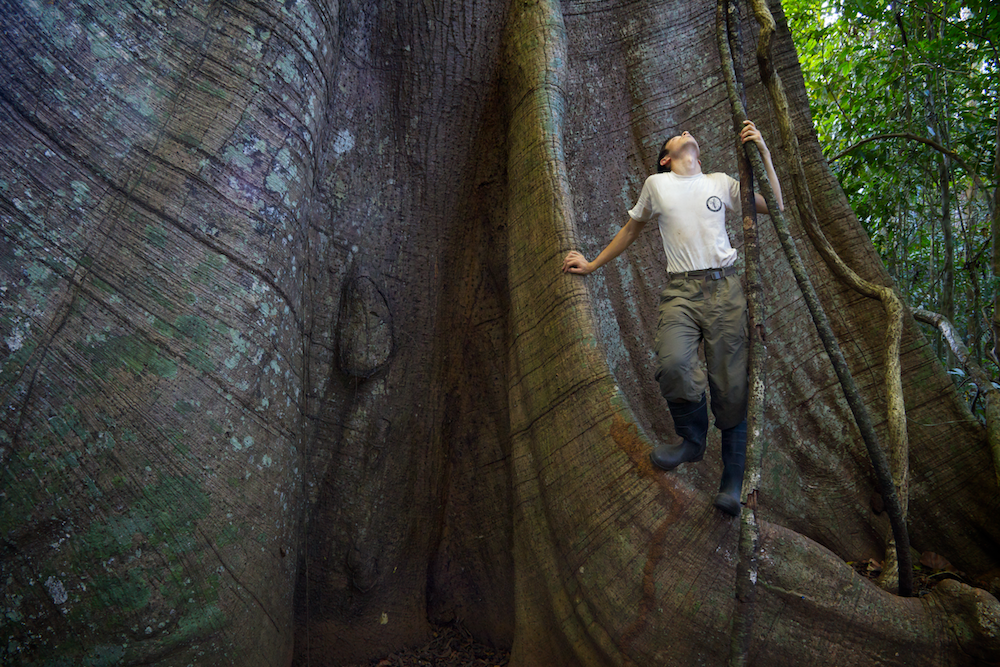

Supplement: Additional file 16 — “This image is of a constructed colony of decoy Northern Gannets on the North Island of New Zealand. Calls are broadcast through solar powered speakers, and the decoys were set up to try to re-establish gannets on a preserved piece of land where Northern Gannets historically were found. I took this photograph while I was participating in a large scale ecological restoration project on this property.” Attribution: Hara Woltz (Columbia University). [file 1472-6785-13-6-S16.png]

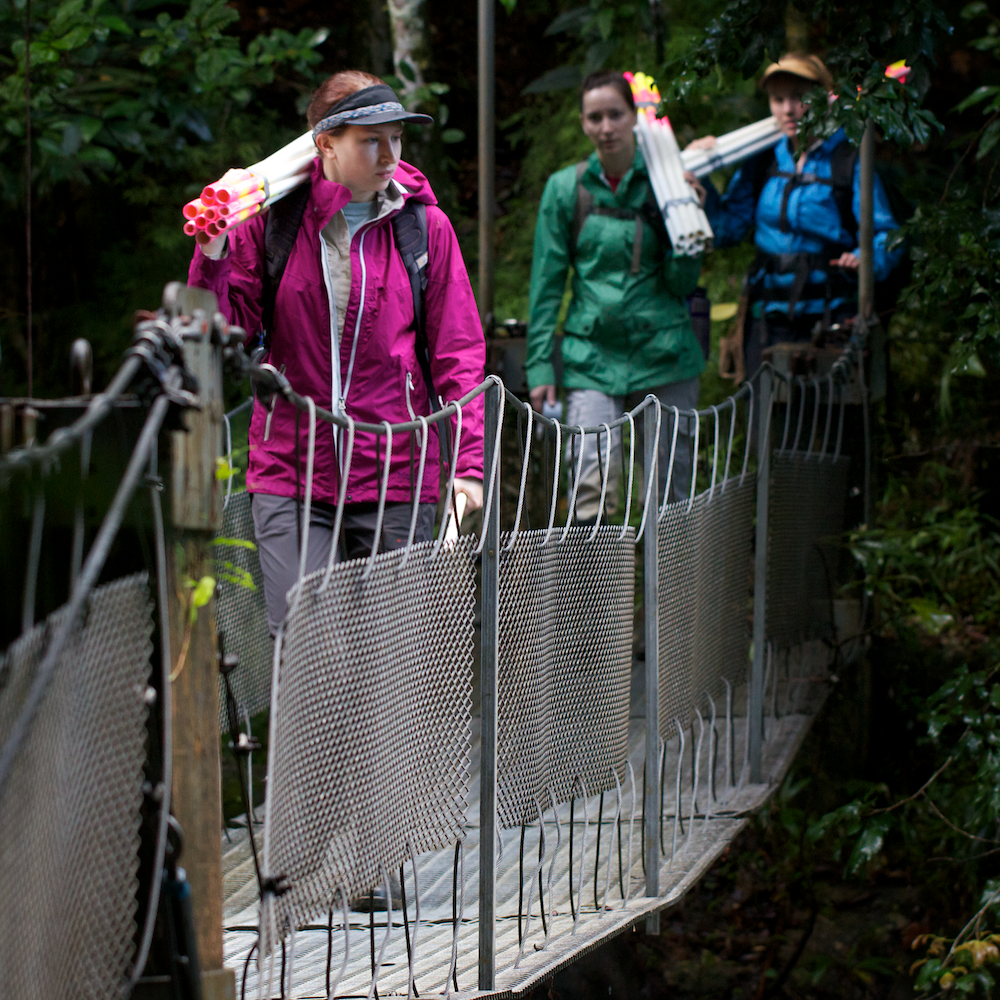

Supplement: Additional file 17 — “Carrying vegetation survey equipment to a forest dynamics plot, Puerto Rico” Attribution: Benjamin Blonder (University of Arizona). [file 1472-6785-13-6-S17.png]

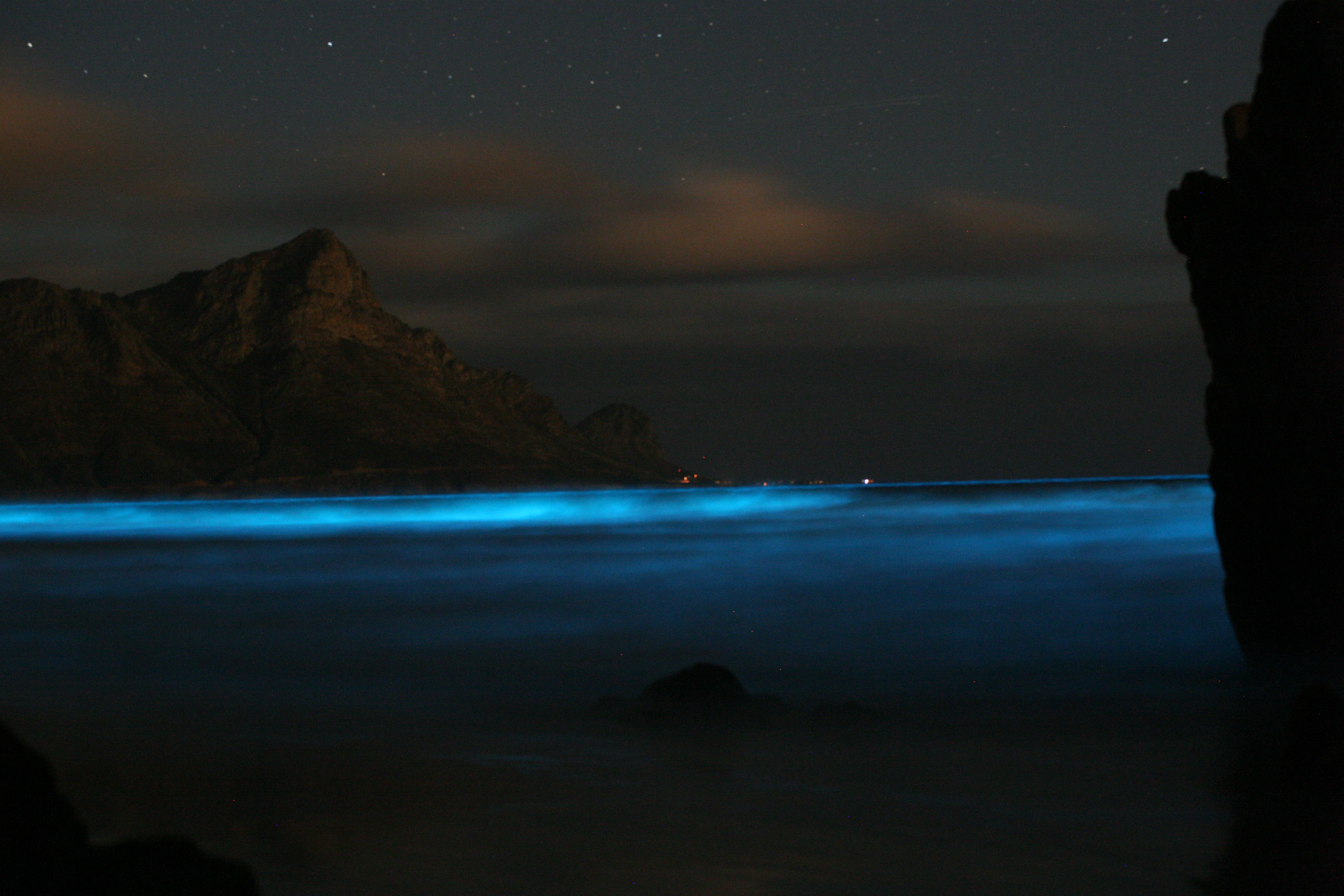

Supplement: Additional file 18 — “Blue Tide. Seasonal winds can cause the upwelling of nutrients which in turn can cause plankton populations to bloom as "red tides." Here, a dinoflagellate population (Noctiluca sp.) turns the ocean a luminous blue colour as the disturbance by the wind triggers a light-generating chemical reaction. The production of light is thought to attract fish predators that prey on potential predators of the dinoflagellates.” Attribution: Bruce Anderson (University of Stellenbosch). [file 1472-6785-13-6-S18.jpeg]

## Slide 1
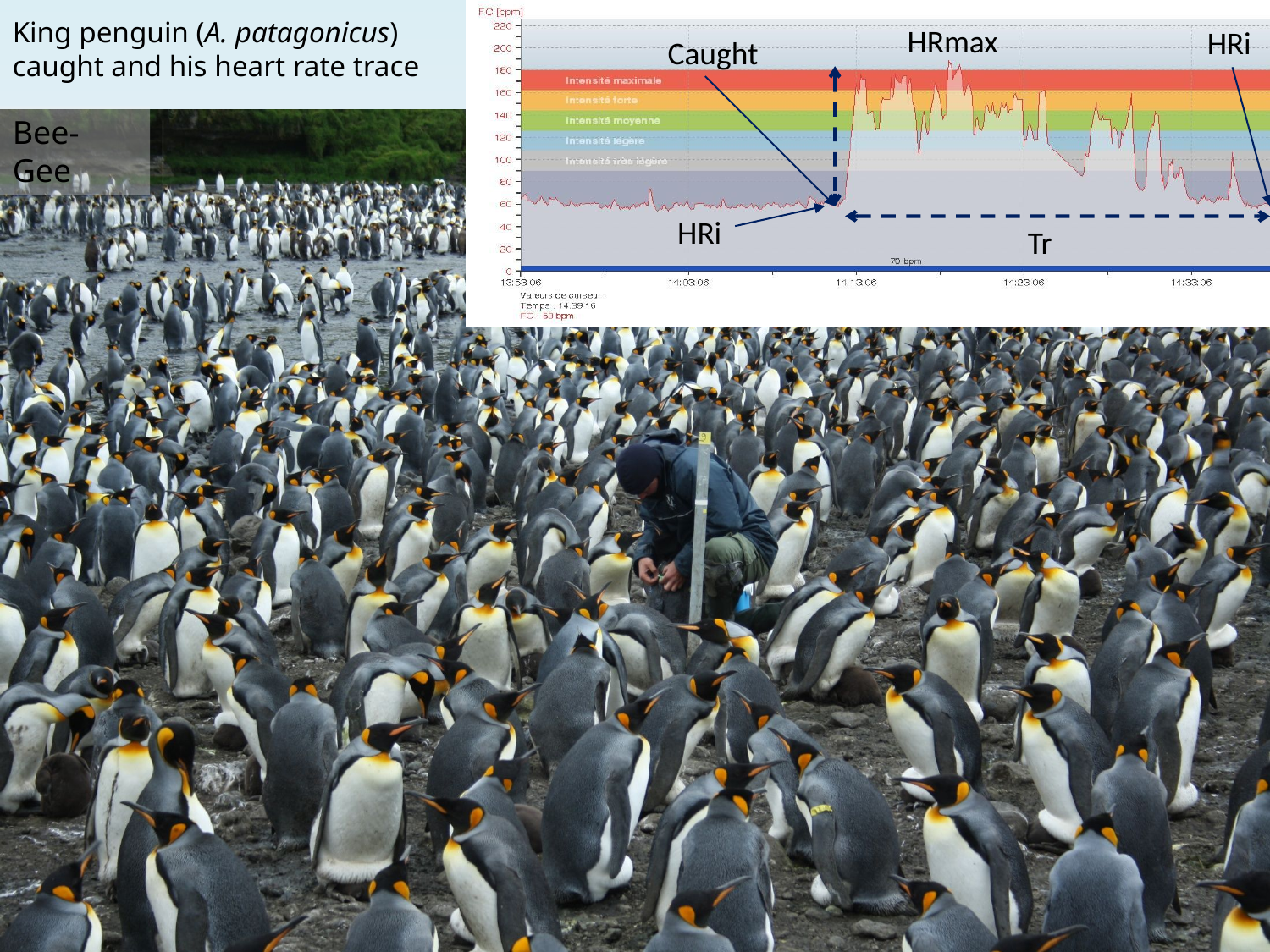

King penguin (A. patagonicus) caught and his heart rate trace
HRmax
HRi
Caught
Bee-Gee
HRi
Tr

Supplement: Additional file 19 — “To well understand all the ecological process that drive the physiology and behavior of animals in the nature, it appears really important to study organisms on the field. It is also important for the scientists to estimate our impact during study on the free living species. Here, we measured the heart rate (HR) excess (the number of heart beats produced in excess of resting HR due to different kind of stress: capture, 10 m approach or sound). To do this, we used an externally mounted-HR logger (Polar® model RS800) on a free long-lived seabird, the king penguin (Aptenodytes patagonicus). We compared the HR response due to the same 3 stressors between two parts of the colony: one disturbed by human presence and one without humans. We show that the HR response is lower for the bird in the non disturbed place, for 10 m-approach and sound stress, but there is no difference between the two places for the stress of capture. Habituation of the king penguin or selection of the bird who can support the human proximity? The picture was taken in Crozet archipelago (46°24′41″S ; 51°45′22″E ), a French island of the austral ocean ; the 13th February 2012. The experimenter was positioning the HR logger on the back of the king penguin. On the right top of the image, we show an example of heart race trace we obtain and we can see clearly when the bird was caught for the stress of capture.” Attribution: Benoit Gineste (Evolutionary EcoPhysiology Group, CNRS). [file 1472-6785-13-6-S19.ppt]
